# CO_2_ Conversion on N-Doped Carbon Catalysts via Thermo- and Electrocatalysis: Role of C–NO_*x*_ Moieties

**DOI:** 10.1021/acscatal.2c01589

**Published:** 2022-08-04

**Authors:** Dorottya Hursán, Marietta Ábel, Kornélia Baán, Edvin Fako, Gergely F. Samu, Huu Chuong Nguyën, Núria López, Plamen Atanassov, Zoltán Kónya, András Sápi, Csaba Janáky

**Affiliations:** † Department of Physical Chemistry and Materials Science, University of Szeged, H-6720 Szeged, Hungary; ‡ Department of Applied and Environmental Chemistry, University of Szeged, H-6720 Szeged, Hungary; § Interdisciplinary Excellence Centre, University of Szeged, H-6720 Szeged, Hungary; ∥ Institute of Chemical Research of Catalonia, The Barcelona Institute of Science and Technology, 43007 Tarragona, Spain; ⊥ Department of Chemical and Biomolecular Engineering, University of California Irvine, Irvine, California 92697, United States; # National Fuel Cell Research Center, University of California Irvine, Irvine, California 92697, United States

**Keywords:** N-doped carbon, CO_2_ reduction, electroreduction, thermal conversion, active center, N-oxide, reaction mechanism, DFT

## Abstract

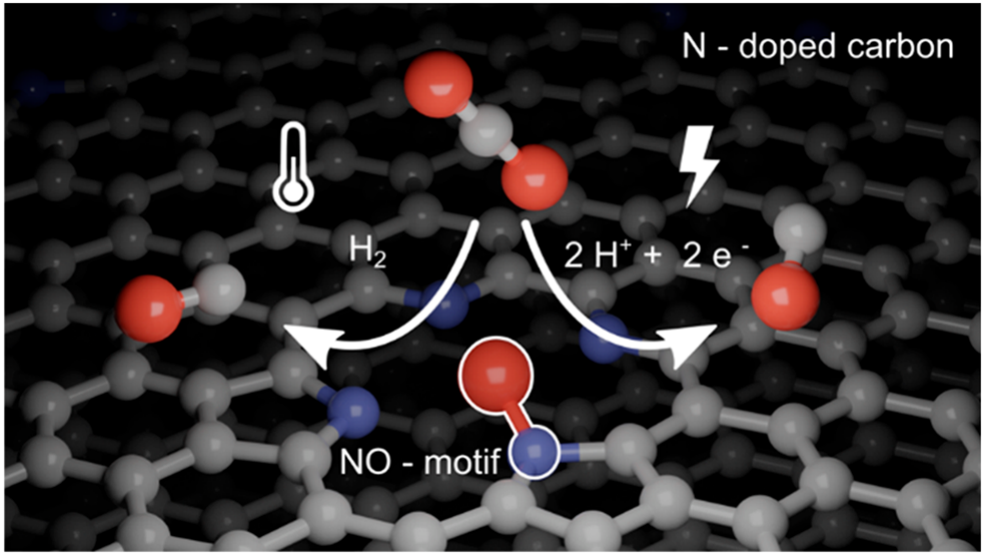

N-doped carbon (N–C) materials are increasingly popular in different electrochemical and catalytic applications. Due to the structural and stoichiometric diversity of these materials, however, the role of different functional moieties is still controversial. We have synthesized a set of N–C catalysts, with identical morphologies (∼27 nm pore size). By systematically changing the precursors, we have varied the amount and chemical nature of N-functions on the catalyst surface. The CO_2_ reduction (CO_2_R) properties of these catalysts were tested in both electrochemical (EC) and thermal catalytic (TC) experiments (i.e., CO_2_ + H_2_ reaction). CO was the major CO_2_R product in all cases, while CH_4_ appeared as a minor product. Importantly, the CO_2_R activity changed with the chemical composition, and the activity trend was similar in the EC and TC scenarios. The activity was correlated with the amount of different N-functions, and a correlation was found for the −NO_*x*_ species. Interestingly, the amount of this species decreased radically during EC CO_2_R, which was coupled with the performance decrease. The observations were rationalized by the adsorption/desorption properties of the samples, while theoretical insights indicated a similarity between the EC and TC paths.

## Introduction

The increasing energy demand and the excessive use of fossil resources has led to a continuous rise in the atmospheric CO_2_ concentration since the industrial revolution. This has resulted in severe alteration of the natural carbon cycle and a significant increase in the annual global temperature in comparison to the preindustrial era.^1^ The conversion of CO_2_ into commodity chemicals and transportation fuels can be a promising solution to supplement the natural carbon cycle and shift the currently fossil-fuel-based energy system to a circular one, relying mainly on renewable energy sources.^2,3^ Different routes exist to convert CO_2_ into valuable materials,^4^ such as thermochemical (TC), electrochemical (EC) and photochemical methods.^5−7^ The TC CO_2_ conversion is the most mature among these,^2,8^ dating back to the beginning of the 20th century when the Sabatier reaction was discovered. Since then, the thermal hydrogenation of CO_2_ is industrially practiced in many countries (e.g., CO_2_ to methanol plants in Iceland, Japan, and Germany).^2^ Recently, EC CO_2_ reduction (CO_2_R) has also attracted great attention, having several advantages in comparison to the thermal process, such as (i) ambient pressure and temperature, (ii) swift integration to renewable energy sources, and (iii) ease of control. Despite the extensive research and significant advancement in this field in recent years, the industrialization of such processes has yet to be realized.^9^

Independently of the source of energy used to drive the CO_2_ conversion, catalysts are the core of these processes. They are indispensable in enhancing reaction rates and controlling selectivity by opening up new reaction pathways.^10^ In both EC and TC CO_2_ conversions, metal-based materials (often precious metals) are the most efficient and selective catalysts. For example, in the TC process, CO can be produced through the reverse water-gas-shift reaction using metals (Pt, Ni, Fe, Pd, etc.) supported on semiconducting oxides (TiO_2_, CeO_2_, etc.) at ambient pressures.^11−13^ The proper combination of reducible oxides with nonprecious metals or spinel structures can also lead to CH_4_ formation during the CO_2_ hydrogenation.^14,15^ In the synthesis of methanol, mainly Cu and Cu-Zn oxide based catalysts are employed.^16^ Producing long-chain hydrocarbons is also in the focus of research, typically employing tandem catalysts.^16−18^ In the EC CO_2_R, the most selective CO-producing catalysts are Au and Ag,^19−21^ while formic acid is mostly obtained on Sn-based materials.^22,23^ Cu is unique in this sense, being the only metal capable of forming alcohols and hydrocarbons with reasonable rates.^24−26^

To make the CO_2_ conversion processes economically viable at scale, less expensive, (precious) metal-free catalysts need to be developed.^27,28^ N–C materials are promising, cost-effective alternatives to traditional catalysts in several electrochemical processes, such as in the oxygen reduction,^29^ hydrogen evolution,^30^ and CO_2_R reactions.^31,32^ In CO_2_R using metal-free N–C catalysts, mainly CO^28,33,34^ and formic acid were produced, but in some cases the formation of alcohols and hydrocarbons has also been reported.^35,36^ In TC CO_2_ conversion reactions, however, N-doped carbons alone are rarely used as catalysts. In one work, Al_2_O_3_-supported N-doped graphene quantum dots were tested in the hydrogenation of CO_2_. CO was the predominant product at lower temperatures (maximum of 85% selectivity at 300 °C), while comparable amounts of CH_4_ and CO were formed above 380 °C.^37^ Similar catalysts were employed in the EC CO_2_R as well, where, in addition to the most common C_1_ products (CO and formate), hydrocarbons and multicarbon oxygenates were also reported.^36^ Recently, a metal-free biomass-derived N-doped graphene catalyst also exhibited CH_4_ formation activity in the thermal conversion.^38^ N-doped carbons have also been applied as a support for metal catalysts. For instance, on a Cu-Zn/NrGO catalyst methanol was produced,^39^ while a Fe/N-CNT catalyst favored the formation of CO and C_1_–C_5_ hydrocarbons.^40^ In all cases, the presence of N atoms in the carbons (particularly pyridinic N) was considered to be important in the process, by helping CO_2_ chemisorption and increasing the metal dispersion.

Identifying the active centers of (M)–N–C catalysts and getting insights into the reaction mechanism are challenging, as the chemical structure of these materials is not well-defined and multiple active centers might be present in one material.^31,41,42^ Moreover, several factors other than the chemical identity of the active sites (e.g., morphology and local environment of active center N-defects as preferential adsorption sites) can play a role in defining the catalytic performance.^31,32,43,44^ Because of these factors, there is still no consensus about the nature of active sites and the role of surface functional groups of the N–C materials in the CO_2_R reaction. Many works have suggested the importance of pyridinic N,^28,45−47^ but pyrrolic N,^48,49^ the partially positive C atoms next to pyridinic N,^50^ and intrinsic carbon defects^51^ (achieved by heteroatom removal) were also suspected. Interestingly, the role of −NO_*x*_ groups is often overlooked in these works, probably because of their significantly lower concentration in comparison to other N-moieties (e.g., pyridinic, pyrrolic). To the best of our knowledge, there has been no previous work that considered the possible participation of these species in the CO_2_R.

As was highlighted above, both EC and TC play a vital role in the transformation of CO_2_ into useful products. A similar mechanism was suggested for the EC CO_2_R to hydrocarbons and the TC modified Fischer–Tropsch (H_2_ + CO_2_) process on certain catalysts;^52,53^ however, these two fields are generally considered as two *separate disciplines*.^54^ In a recent work, the activity of the same Ni–N–C catalyst was compared in the EC CO_2_ reduction and the reverse water-gas-shift reaction. Through kinetic investigations, an analogous reactivity was found in the two processes; however, the absolute reaction rates were ca. 25–50 times higher in the EC CO_2_ reduction. This difference was attributed to the lower energy barrier imposed on the EC system.^55^

Motivated by the above findings, we aimed to find out whether the same structural factors determine the catalytic activity in EC and TC CO_2_R processes on N–C catalysts, or there are other factors to consider. In other words, can we have a general conclusion about the role of the structural features of an efficient catalyst in the CO_2_ conversion process? Here, we analyze the two scenarios with a systematic comparison of the performance of the same set of highly porous N-doped carbon materials in EC and TC CO_2_R. To uncover the role of surface functional groups, the chemical composition of the catalysts was varied by changing the precursor material and employing chemical post-treatments. After characterizing the pore structure of the materials (employing N_2_ adsorption/desorption and electron microscopy) and their chemical composition (by X-ray photoelectron spectroscopy), we tested their catalytic activity in the TC and EC CO_2_ conversion. To explain the observed trends, the CO_2_ adsorption characteristics of the materials were investigated using temperature-programmed desorption and quartz-crystal microbalance techniques. Our experimental studies were complemented with DFT calculations, to gain an atomistic understanding of the observed trends.

## Experimental Section

### Catalyst Synthesis

N–C catalysts were synthesized from conjugated polymer precursors using a sacrificial support method.^32^ The effect of the pore structure was deconvoluted by fixing the pore size at ∼27 nm, which was proved to be favorable in the EC CO_2_R.^32^ Polyaniline (PANI), polypyrrole (PPy), poly(*o*-phenylenediamine) (PoPD), and mixtures of PANI and PoPD were used as the precursors. In a typical synthesis, 52.3 cm^3^ solution containing 0.58 M of the respective monomer, 0.72 M hydrochloric acid (HCl, VWR International, 37%), and 0.18 g cm^–3^ of silica nanoparticles (LUDOX-TM50, Aldrich, 27 nm mean diameter) was vigorously stirred for 15 min to adsorb the monomer molecules on the surface of the silica particles. Then, 12 cm^3^ 2.7 M ammonium persulfate (APS; (NH_4_)_2_S_2_O_8_; Acros Organics) oxidant in 1 M HCl (to avoid any possible transition-metal contamination) was added dropwise to the monomer solution at 0 °C (ice bath). The mixture was stirred for 24 h to complete the polymerization process. The molar ratio of the monomer relative to the oxidant was 0.8. The obtained polymer/SiO_2_ composites were freeze-dried and then pyrolyzed at 900 °C in a tube furnace under N_2_ flow (110 cm^3^ min^–1^). The heating program was as follows: RT–ramp 5 °C min^–1^–80 °C (1 h)–ramp 5 °C min^–1^–900 °C (2 h). The silica nanoparticles were etched out overnight with an excess amount of 15 wt % HF (VWR, 50 wt %) solution. Finally, the N–C catalysts were washed thoroughly with ultrapure water and vacuum-filtered, until close to neutral pH (>5) of the supernatant solution was reached. For the N–C samples prepared from mixtures of PoPD and PANI, the polymers were mixed in a mortar in molar ratios of 30:70 and 70:30 after freeze-drying. The obtained samples are denoted as PoPD-C, PANI-C, PPy-C, PANI(30)-PoPD(70)-C, and PANI(70)-PoPD(30)-C, referring to the polymer precursors.

In case of the PoPD-C catalyst, different postchemical treatments were also employed to further tune the chemical composition and/or porosity. During the KOH treatment, the PoPD-C sample was mixed with 7 M KOH (*m*(KOH)/*m*(N–C) = 3), dried under vacuum at 60 °C, and subjected to a second heat treatment at 800 °C. Heating program: RT–ramp 5 °C min^–1^–800 °C (1 h). During the NH_3_ treatment the PoPD-C sample was heat treated under an NH_3_ flow (70 cm^3^ min^–1^) at 900 °C for 1 h. The heating and cooling steps were performed under flowing N_2_. After these second heat treatment steps in the reactive environments, the catalysts were again washed thoroughly with ultrapure water. The KOH and NH_3_ post-treated samples are denoted as PoPD-C-KOH and PoPD-C-NH_3_, respectively.

Catalysts were spray-coated onto preheated (110 °C) glassy-carbon plates, using custom-designed automated spray-coater equipment. Before spray-coating, the substrates were polished with 0.05 μm MicroPolish Alumina (Buehler), rinsed and sonicated in acetone (C_3_H_6_O, VWR), ethanol (C_2_H_6_O, 99%, VWR), and ultrapure water. The catalyst suspension contained 5 mg mL^–1^ N–C catalyst and 100 μL Nafion dispersion (FuelCell Store, 10 V/V%) in 10 mL ethanol–water mixture (50 V/V%). The exact amount of catalyst coated was always weighed with a microbalance.

### Catalyst Characterization

Transmission electron microscopy (TEM) images were recorded using a FEI Tecnai G2 20 X-Twin Type instrument, operating at an acceleration voltage of 200 kV. For morphology studies a Hitachi S4700 field emission scanning electron microscope (SEM) was used, which was operated at an acceleration voltage of 10 kV. Raman spectra were recorded with a Senterra Compact Raman microscope (Bruker), using a 532 nm laser excitation wavelength, at a power of ≤2.5 V mW and a 50× objective. N_2_ adsorption/desorption isotherms were recorded at 77.4 K on a Quantachrome Nova 3000e instrument. Samples were degassed at 200 °C for 2 h before measurement. Pore size distribution curves were calculated using the Barrett–Joyner–Halenda method excluding points below 0.35 relative pressure. X-ray photoelectron spectroscopy (XPS) was performed with a SPECS instrument equipped with a PHOIBOS 150 MCD 9 hemispherical analyzer. The Al Kα radiation (*h*ν = 1486.6 eV) of a dual-anode X-ray gun was used as an excitation source and operated at 150 W power. Spectra were recorded in a fixed analyzer transmission mode with 40 eV pass energy for the survey and 20 eV pass energy for the high-resolution spectra. No charge neutralization was needed. For the fitting, a Shirley background was employed in the case of the N 1s spectra and a Tougaard background for the C 1s spectra. Peaks were fitted with a Gaussian–Lorentzian (30) line shape, except for the graphitic carbon, which was fitted with an asymmetric line shape. Peak widths were constrained to 1.3–1.6 eV.

Temperature-programmed desorption (TPD) of CO_2_ was performed with a BELCAT-A apparatus using an externally heated reactor (quartz tube with 9 mm outer diameter). The catalyst samples were treated in Ar at 300 °C for 30 min before the measurements. Thereafter, each sample was cooled under flowing Ar to 50 °C and, after purging of the samples with 10% of CO_2_ in Ar, the sample was heated linearly at a rate of 5 °C min^–1^ from 100 to 500 °C under an inert atmosphere, while the CO_2_ desorption was monitored by a thermal conductivity detector (TCD). The CO_2_ adsorption characteristics of the catalyst layers were studied with an SRS QCM-200 quartz-crystal microbalance (5 MHz resonant frequency). The catalyst loading on the QCM crystals was 50 μg cm^–2^. During a typical measurement, N_2_ and CO_2_ were periodically introduced into the QCM cell (in 30 min cycles) and the frequency change of the crystal was recorded. The mass changes related to the CO_2_ adsorption/desorption were calculated from the measured frequency changes using the calibration constant of the crystal.

### Electrochemical Methods

Electrochemical data were acquired using an Autolab PGSTAT 204 potentiostat/galvanostat. Potentials were measured against an Ag/AgCl/3 M NaCl reference electrode but are presented versus the reversible hydrogen electrode (RHE) throughout the text (*E*_RHE_ = *E*_Ag/AgCl_ + 0.20 V + 0.059 V × pH). The counter electrode was Pt foil in all experiments. Our control experiments with glassy-carbon counterelectrodes demonstrated that the possible contribution of Pt contamination to the electrochemical activity of the working electrodes can be safely excluded (Figure S8).

The electrochemically active surface area of the catalyst-coated glassy-carbon electrodes was determined from cyclic voltammetry. Cyclic voltammograms (CVs) were recorded in a one-compartment three-electrode cell, using an Ar-purged 1 M sodium sulfate solution (Na_2_SO_4_ anhydrous; 99% Alfa Aesar) as the electrolyte. Roughness factors of the N–C electrodes were estimated from the double-layer capacitance values (*Q*_dl_) determined from the CVs, recorded between 0.21 and 0.61 V (vs RHE) with different sweep rates. The double-layer current (*I*_dl_) was determined at 0.41 V as the difference between the anodic and cathodic currents (*I*_dl_ = *I*_a_ – *I*_c_). *I*_dl_ was plotted against the sweep rate (*v*), and *Q*_dl_ was calculated from the slope (*s*) of this curve (*Q*_dl_ = *s*/2). The *Q*_dl_ values obtained for the N–C electrodes were compared to that of a bare, smooth glassy-carbon electrode (assuming a surface roughness of 1). Roughness factors were given by the ratio of these two values.

The electrocatalytic activity of the catalyst samples was tested by linear sweep voltammetry and chronoamperometry in a two-compartment sealed electrochemical cell. The cathode and anode compartments were separated by a Nafion-117 membrane. For the analysis of the gas-phase products, the cathode compartment of the cell was directly connected to the inlet of the gas chromatograph via a six-port valve (see details below). In the CO_2_R experiments we used CO_2_-saturated (Messer; 99.995%) potassium hydrogen carbonate (KHCO_3_; VWR) electrolytes. The error bars on the figures reflect the standard deviation of three parallel measurements on separate electrodes.

### CO_2_ Reduction Product Analysis

Gas-phase CO_2_ reduction products were analyzed by online gas chromatography (GC), using a Shimadzu-2010 Plus GC instrument equipped with a barrier ionization discharge (BID) detector. For the separation, a Shincarbon-ST column was used. During electrolysis, 0.5 mL of headspace gas was injected into the GC at around 15, 40, and 68 min. Analysis parameters were as follows: carrier gas, helium; oven program, 35 °C (2.5 min)–20 °C min^–1^–270 °C (3 min); injection temperature, *T* = 150 °C; linear velocity was controlled by the pressure 250 kPa (2.5 min)–15 kPa min^–1^–400 kPa (7.5 min); split ratio:10.

Liquid-phase products were analyzed by NMR spectroscopy. Spectra were acquired on a Bruker NMR Advance 500 MHz instrument. Water suppression was employed to eliminate the peak of the solvent. For the NMR measurement, 450 μL of the electrolyte sample was mixed with 50 μL D_2_O (Sigma-Aldrich; 99.9 atom % D) containing dimethyl sulfoxide (DMSO, C_2_H_6_SO; Alfa Aesar) and phenol (C_6_H_6_O; Sigma-Aldrich) as the internal standards. The ratio of peak areas of the products and the internal standards was used for the calibration. Peaks located right of the water signal were compared to the peak area of DMSO, while signals of products left of the water signal were compared to the peak area of the phenol.

### TC Hydrogenation of CO_2_ in a Continuous-Flow Reactor

The catalytic reactions were performed in a fixed-bed continuous-flow reactor (200 mm long with 8 mm inner diameter), which was heated externally. The dead volume of the reactor was filled with quartz beads. The operating temperature was controlled by a thermocouple placed inside the oven close to the reactor wall, to ensure precise temperature measurements. For catalytic studies, small fragments (about 1 mm) of slightly compressed pellets were used. Typically, the reactor filling contained 150 mg of catalyst. The CO_2_:H_2_ mixture was introduced with the aid of mass flow controllers (Aalborg). The reacting gas flow entered and left the reactor through an externally heated tube to avoid condensation of the products. The analysis of the products and reactants was performed with an Agilent 4890 gas chromatograph using a Porapak QS packed column connected to a thermal conductivity detector and an Equity-1 column connected to a flame ionization detector.

Before the catalytic experiments, catalysts were pretreated in Ar at 300 °C for 30 min to remove any adsorbed species from their surface, followed by a reaction test immediately between 300 and 700 °C. The CO_2_:H_2_ molar ratio was 1:4, and the flow rate of such mixture was 20 mL min^–1^ balanced with 20 mL min^–1^ of Ar, resulting in a total flow rate of 40 mL min^–1^. During the reaction, the temperature was increased in 50 °C steps, with a 10 °C min^–1^ heating rate. After 15 min of equilibration at each temperature, gas samples were analyzed with GC before the next heating step was started. At 700 °C, a 300 min time-on-stream test was performed for all catalysts. Activation energy calculations were performed in the range of 550–700 °C with <15% maximum conversion based on the Arrhenius-plot fitting method.

### Computational Details

The reaction profiles were investigated with atomistic simulations with the VASP 5.4.4 code.^56−59^ The functional of choice was PBE^60^ with van der Waals interactions being included via DFT-D3.^61^ Inner electrons were represented by PAW pseudopotentials,^62,63^ while valence monoelectronic states were expanded with plane waves with a maximum energy cutoff energy of 450 eV. Bulk calculations were performed with 3 × 3 × 3 *k*-point sampling and slab calculations with 3 × 3 × 1, including dipole correction. The N–C catalysts were represented by a hexagonal supercell (6 × 6) comprised of a single layer of graphite. All structures are available on the ioChem-BD database:^64^ DOI: 10.19061/iochem-bd-1-199.

## Results and Discussion

### Catalyst Synthesis and Characterization

We synthesized the metal-free porous N–C catalysts from conducting polymer precursors by a sacrificial support method.^32^ The precursors were polyaniline (PANI), polypyrrole (PPy), poly(*o*-phenylenediamine) (PoPD) and mixtures of PANI and PoPD in 30:70 and 70:30 molar ratios. First, the respective monomers were chemically polymerized in the presence of monodisperse silica colloids (27 nm mean diameter), functioning as templates for pore formation. Then, the obtained polymer/silica composites were pyrolyzed under an N_2_ flow at 900 °C. Finally, the silica nanoparticles were etched out with HF solution. To study the effect of postchemical treatments on the CO_2_R activity, we also synthesized a KOH- and NH_3_-treated catalyst, starting from PoPD-C.

**Figure 1:**
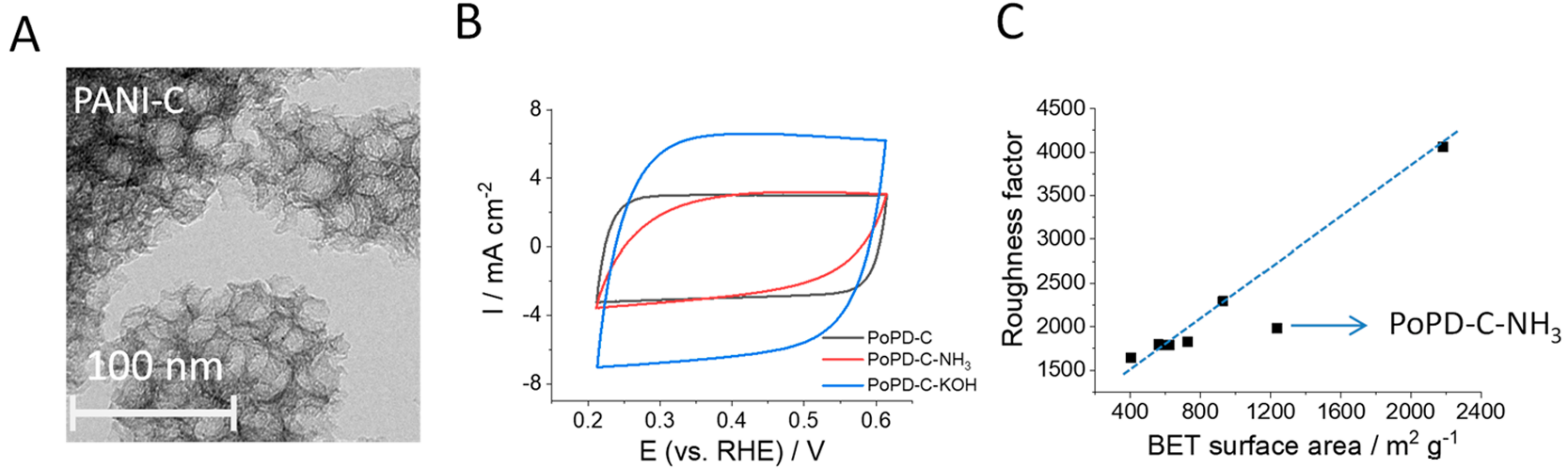
(A) TEM image of PANI-C. (B) Cyclic voltammograms of PoPD-C, PoPD-C-NH_3_, and PoPD-C-KOH (mass loading: 1.00 mg cm^–2^) measured in an Ar-purged 1 M Na_2_SO_4_ solution with a 50 mV s^–1^ scan rate. (C) Correlation between the BET surface area of the powder samples and the roughness factor (determined by cyclic voltammetry) of the N–C electrodes.

TEM images in Figure 1A and Figure S1 show that all catalysts have an interconnected carbon structure. In the case of the samples prepared without postchemical treatments, mesopores, formed during the template-assisted synthesis, were observed. By an analysis of multiple images the average pore sizes were determined, which reflect the mean diameters of the silica nanoparticles (PPy-C, 26.2 ± 2.9 nm; PANI-C, 26.7 ± 2.7 nm; PANI(30)-PoPD(70)-C, 26.7 ± 2.5 nm; PANI(70)-PoPD(30)-C, 27.3 ± 2.6 nm; PoPD-C. 26.4 ± 3.9 nm). In the structure of PoPD-C-NH_3_ catalyst, the mesopores are still present (25.2 ± 3.3 nm); however, those are hardly observable in case of PoPD-C-KOH, as they probably collapsed during the KOH treatment. The pore structure was further analyzed by measuring the N_2_ adsorption/desorption isotherms and deriving the pore size distribution (PSD) curves (Figures S2 and S3**)**. The isotherms with the hysteresis loops are characteristic of mesoporous materials, with additional microporous features in the case of PoPD-C-NH_3_ and PoPD-C-KOH (higher N_2_ uptake at low relative pressures). The PSD curves show a maximum between 20 and 30 nm in the case of the untreated catalysts, though they suggest a wider distribution than those determined from the TEM images. For PoPD-C-NH_3_, this peak was less pronounced in comparison to the bare PoPD-C. At the same time, a maximum developed below 5 nm, indicating that smaller mesopores were formed during the NH_3_ treatment. In the case of PoPD-C-KOH, however, there was no definite maximum in the mesopore range, in accordance with the TEM analysis. We also determined the BET surface areas from the isotherms, which are presented in Table S1. The specific surface areas varied between 400 and 1000 m^2^ g^–1^ for the untreated samples and increased in the order of PPy-C < PANI-C < PoPD-C. The BET surface areas of PANI(30)-PoPD(70)-C and PANI(70)-PoPD(30)-C fell between those of the catalysts prepared from pure PANI and PoPD, as expected. Although we used the same monomer/silica molar ratios during the synthesis in each case, the different polymerization and carbonization yields could result in different surface structures and therefore surface areas. With the NH_3_ treatment the surface area increased by ca. 30%, while the KOH treatment doubled the surface area of the bare PoPD-C. In the latter case, the collapse of the mesopores was accompanied by micropore formation, which is in accordance with earlier literature reports.^65^

To determine the roughness factors of the electrodes, CV scans were recorded in a potential range where no Faradaic process takes place (Figure 1B and calculated roughness factors in Table S1**)**. The correlation between the electrochemically measured roughness factors and the BET surface areas is presented in Figure 1C. Only the PoPD-C-NH_3_ catalyst stands out from the linear trend. In this case, the electrochemically determined roughness factor was lower than what would be expected from the BET surface area. Comparing the CVs of PoPD-C and PoPD-C-NH_3_ (Figure 1B), one can notice that the shape of the voltammogram is distorted from the ideal rectangular form (i.e., typical of capacitive behavior) in case of the NH_3_-treated catalyst. This suggests that this sample contains more abundant defect sites, resulting in lower conductivity, which was further supported by an EIS analysis (Figure S4). The charge transfer resistance was approximately 1 order of magnitude higher for PoPD-C-NH_3_, in comparison to the bare PoPD-C. We employed Raman spectroscopy to characterize the carbon structure of the N–C catalysts (Figure S5). The characteristic D and G bands of carbon materials appeared at ca. 1330 and 1580 cm^–1^, respectively. The intensity ratios of the two bands (*I*_D_/*I*_G_) were between 0.87 and 0.97 for all catalysts and were lower for the PoPD-derived samples (Table S5).

**Figure 2:**
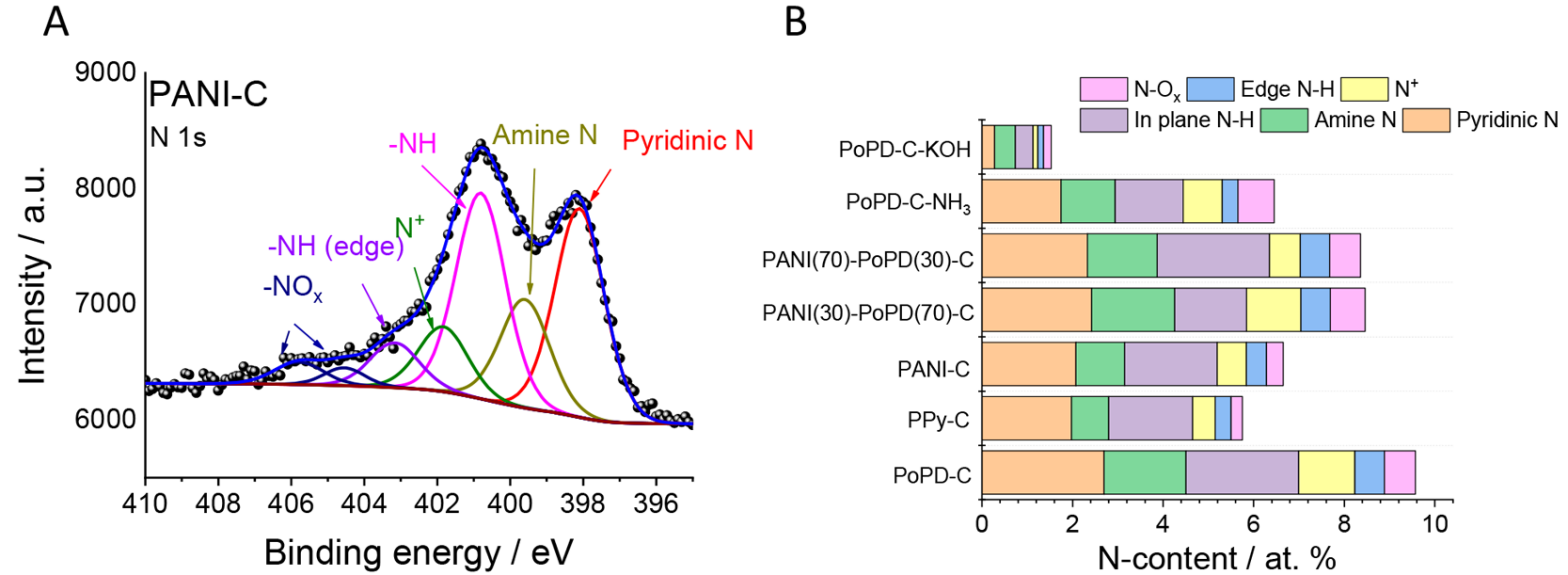
(A) High-resolution N 1s XPS spectrum of PANI-C. (B) N-speciation of the catalysts, determined from the fitting of the high-resolution N 1s spectra.

The surface chemical composition of the catalysts was studied by X-ray photoelectron spectroscopy (Figure 2, Figures S6 and S7, and Tables S3 and S4). The survey spectra (Figure S6) revealed the presence of carbon, nitrogen, and oxygen atoms as the main constituents, and *no trace metal contamination was detected.* The N-content of the untreated N–C catalysts correlated with that of the precursor polymers: PoPD-C had roughly a 2 times higher N-content than PPy-C and PANI-C. During the NH_3_ treatment, the N-content of the bare PoPD-C decreased by approximately 30%, in accordance with previous reports on similarly synthesized catalysts.^66^ The KOH treatment, however, resulted in a drastic (85%) loss in the N-content. We assume that the employed post-treatments etched the carbon matrix, and in turn, a more defect-rich structure formed. The high-resolution N 1s spectra (Figure 2A and Figure S7) could be deconvoluted into six peaks, in accordance with previous literature reports on similar materials,^67,68^ which are pyridinic N (398.12 eV), amine N (399.81 eV), in-plane N–H (400.8 eV), N^+^ (402.00 eV), edge N–H (403.3 eV), and −NO_*x*_ (405.00 and 406.63 eV). As presented in Figure 2B and Table S4, the N-speciation also changed with the varying precursors and post chemical treatments. Among the samples prepared from pure precursors, PPy-C had the highest ratio of pyridinic N, while PoPD-C was the richest in N^+^ moieties. Both post treatments increased the ratio of −NO_*x*_ groups, in comparison to the bare PoPD-C. In the case of PoPD-C-NH_3_, not only the relative, but also the absolute amount of −NO_*x*_ groups increased.

### Electrochemical CO_2_ Reduction Activity

**Figure 3:**
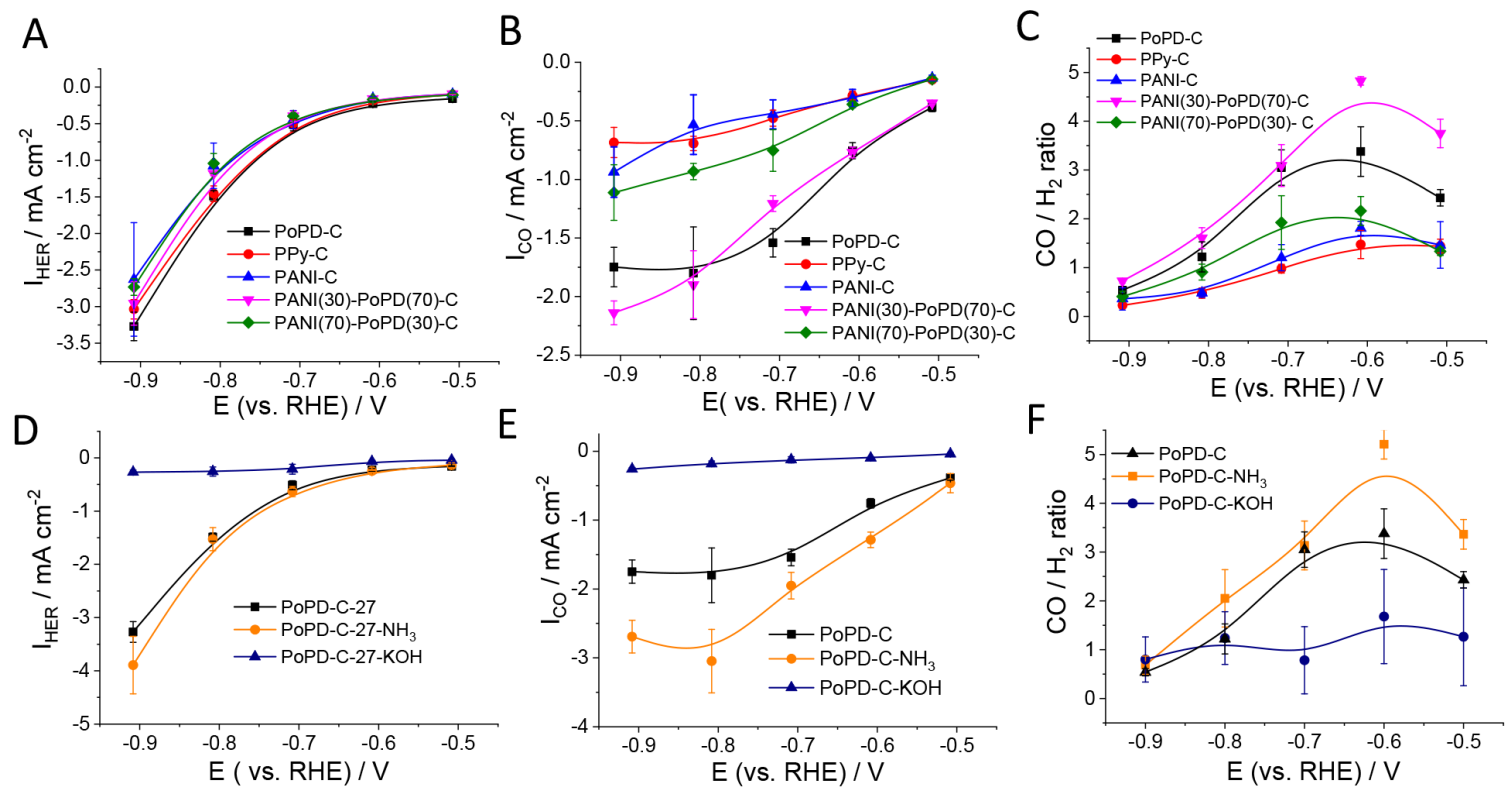
Partial current densities for H_2_ evolution (A, D) and CO-formation (B, E) on the N–C catalysts. Molar ratios of the CO and H_2_ produced (C, F) during the potentiostatic electrolysis at different electrode potentials. The lines through the data points serve only as guides for the eyes. The error bars represent the standard deviation of three measurements, performed on separate electrodes.

First, we tested the EC CO_2_R activity of the N–C electrodes by recording linear sweep voltammograms in a CO_2_-saturated KHCO_3_ electrolyte (Figure S9). The currents increased in the order PANI-C < PPy-C < PANI(70)-PoPD(30)-C < PoPD-C < PANI(30)-PoPD(70)-C among the untreated catalysts. The NH_3_ treatment slightly increased the voltammetric currents, in comparison to PoPD-C. In contrast, the KOH-treated sample showed around 5 times lower currents (at −1.0 V) in comparison to the bare PoPD-C, despite the doubled surface area. The onset potentials (defined as the potential at which the slope of the current–potential curve deviates from zero), were determined from the derivative curves of the voltammograms (Figure S10 and Table S2**)**. This followed the same order as the current densities, being significantly more negative for PoPD-C-KOH, in comparison to the other catalysts. The CO_2_R selectivity was measured during potentiostatic electrolysis between −0.5 and −0.9 V (vs RHE). Stationary currents (total current densities) followed a trend similar to that observed during the LSV measurements (Figure S9). Products formed in the gas and liquid phases were analyzed by *online* gas chromatography and *ex situ* NMR spectroscopy, respectively. The main reduction products were CO and H_2_, while CH_4_ was formed in minor amounts in the gas phase as well. The CO:H_2_ molar ratio was highest at −0.6 V for all studied samples (Figure 3 C,F). Overall, among the catalysts synthesized from different precursors (without post chemical treatment), the highest CO selectivity was achieved for PANI(30)-PoPD(70)-C with 82 ± 2% FE, followed by PoPD-C (76 ± 3%) and then PANI(70)-PoPD(30)-C (64 ± 4%), PANI-C (66 ± 5%), and PPy-C (59 ± 5%) with similar selectivities. The NH_3_-treated catalyst, in addition to the increased current density, showed a slightly higher CO selectivity (83 ± 2%), in comparison to the bare PoPD-C. With the KOH treatment, however, both the reduction currents and the CO_2_R selectivity dropped (59 ± 12% CO at −0.6 V).

To compare the rate of CO_2_R and H_2_ evolution on the different N–C electrodes, we plotted the partial current densities (*I*_CO_ and *I*_HER_) as a function of the electrode potential (Figure 3). Interestingly, the *I*_HER_ values were very similar for all catalysts, except for PoPD-C-KOH, for which it remained below −0.5 mA cm^–2^ in the studied potential regime. The overlapping *I*_HER_ curves suggest similar active sites for the hydrogen evolution reaction for the different catalysts. In contrast, there are significant differences in the CO partial currents. On the basis of the *I*_CO_ values, the untreated catalysts can be categorized into two groups. *I*_CO_ was higher for the samples for which the precursors were rich in PoPD (i.e., PoPD-C and PANI(30)-PoPD(70)-C). For the other three samples, *I*_CO_ was roughly half of the former values, being the highest for PANI(70)-PoPD(30)-C among them. We also normalized the partial currents by the roughness factors of the electrodes (Figure S11), and the order in the current densities remained the same. This indicates that *chemical* differences are responsible for the different catalytic activity, not the varying surface areas. In addition to CO and H_2_, CH_4_ also formed during CO_2_R (Figure S12). Although its Faradaic efficiency remained below 0.5% in all cases, it is still notable, as the formation of hydrocarbons on *metal-free* carbon materials has seldom been reported.

### Thermal Hydrogenation of CO_2_

**Figure 4:**
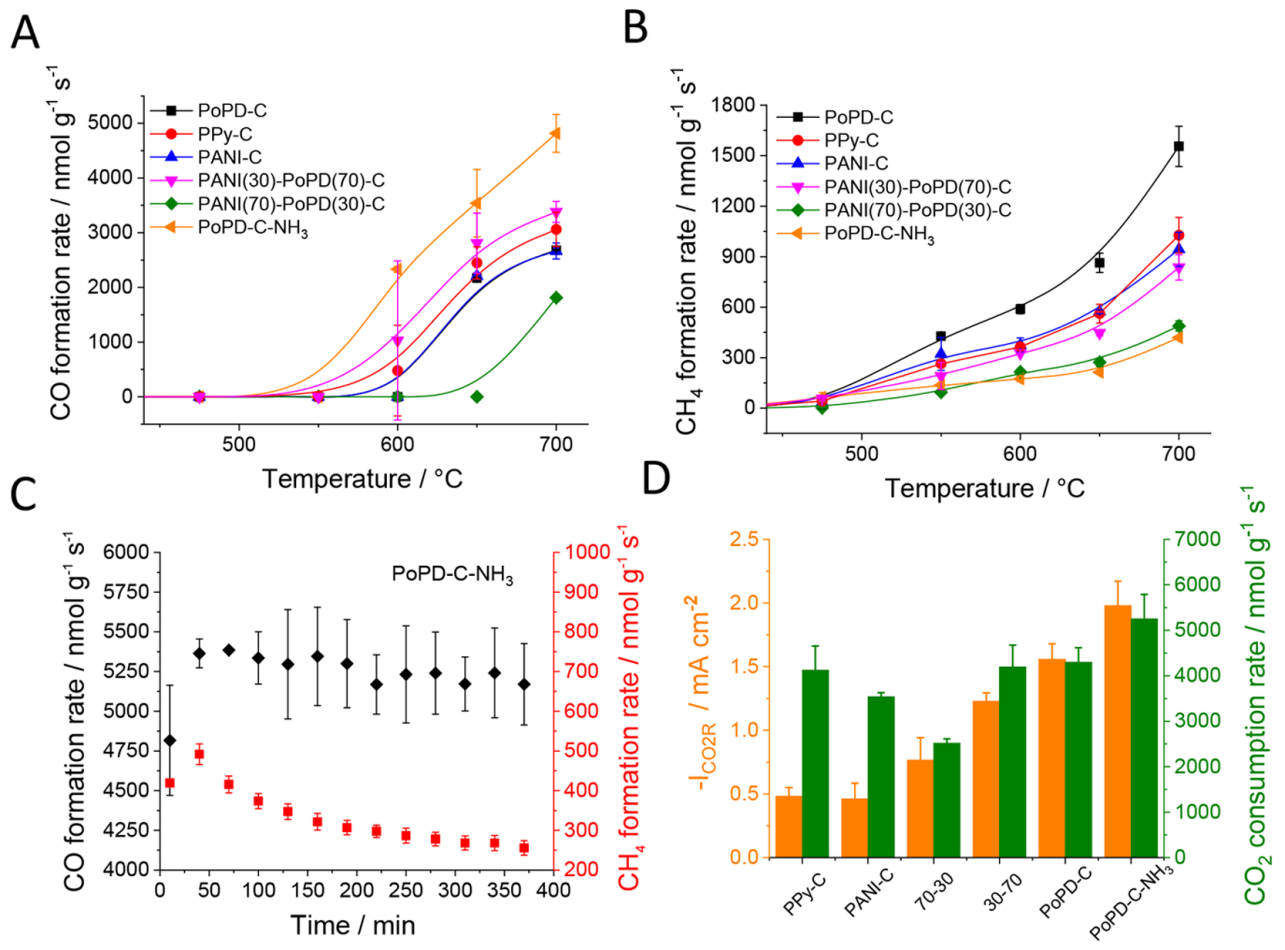
Formation rates of CO (A) and CH_4_ (B) on the studied N–C catalysts as a function of temperature. (C) Stability of the thermal CO_2_ conversion process at 700 °C for the PoPD-C-NH_3_ catalyst. (D) CO_2_ consumption rates in the TC CO_2_ conversion at 700 °C and CO_2_R partial current densities in the EC reduction (at −0.7 V) on the studied catalysts. The error bars represent the standard deviation of three measurements, performed on separate samples from different synthesis batches. The lines along the data points serve only as guides for the eyes.

We also studied the activity of the N–C catalysts in the TC hydrogenation of CO_2_. The PoPD-C-KOH catalyst was excluded from this study, as its pore structure largely differed from the other samples. Furthermore, the thermal reduction of CO_2_ over K-based oxides and hydroxides is well-known from the late 1980s through the CO_2_^–^ formation process,^69^ being very different from the metal-free scenario. TC studies were performed between 300 and 700 °C, where CO and CH_4_ were formed as products, similarly to the EC reaction (Figure 4 A,B). The CO formation started at 600 °C, while the CH_4_ formation started at a slightly lower temperature, 550 °C. The CO selectivity was above 60% in all cases and slightly increased with increasing temperature. After an initial activation period, the catalysts were stable at 700 °C for 400 min toward the formation of CO, but the methane production decreased over time (Figure 4C and Figure S13). The formation rate of CO was usually 1 order of magnitude higher than that of CH_4_. At the same time, the CH_4_:CO molar ratio was also around 10 times higher in the TC than in the EC scenario. While PoPD-C-NH_3_ was the most active catalyst for CO production, in CH_4_ formation PoPD-C was the champion. The relative activities of the samples showed a pattern similar to what was seen in the EC reaction (Figure 4D), except for the higher relative activity of PANI-C and PPy-C in the TC CO_2_ conversion. The reason for this slight deviation can stem from the very different reaction environments (i.e., gas phase vs liquid phase). For example, the dissimilarity of reactant adsorption in the liquid and gas phase may affect the TC and EC processes in a different manner. Nevertheless, the overall similarity suggests that similar active sites take part in the CO_2_ conversion, independently of being thermally or electrochemically activated. The activation energy of CO formation in the thermal process was calculated from the temperature dependence of the reaction rates using an Arrhenius plot (Table S2). In the EC reaction the activation energy is correlated with the onset potential of the Faradaic processes, which was determined from the first derivatives of the LSV curves (Figure S10). We could see only slight differences in both values among the different N–C samples (Table S2), suggesting that the nature of the active centers is similar in the studied materials and only their density varies.

### Active Sites in CO_2_ Conversion

**Figure 5:**
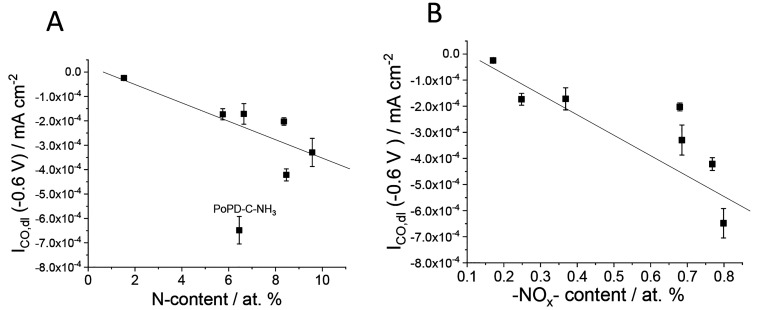
Correlation between the surface chemical composition and the EC CO_2_ reduction activity of the catalysts: (A) total N content; (B) −NO_*x*_ content. The error bars represent the standard deviation of three measurements, performed on separate electrodes.

To unravel the chemical nature of the active sites in the CO_2_R processes, we correlated the amount of different N-species in the catalysts with their CO_2_R performance. With an increase in total N-content, the roughness factor normalized CO partial current densities (*I*_CO,dl_) increased, with the only exception being PoPD-C-NH_3_ (Figure 5A). Interestingly, this catalyst, despite the lower total N-content and the smaller electrical conductivity (vs PoPD-C), showed significantly increased catalytic activity. This suggests that the concentration of the active sites increased because of the NH_3_ treatment. Hence, we plotted the partial current density of CO versus the concentration of the different N-species in the catalysts as well. *Only the −NO*_*x*_*content showed correlation with the I*_*CO*_*value* (Figure 5B). For the other types of N-moieties, there was either no correlation at all or the PoPD-C-NH_3_ was an outlying point (Figure S14). This observation points to the essential role of −NO_*x*_ groups in CO_2_R processes on these N–C materials. The not perfectly linear correlation, however, suggests that other N-moieties, such as pyridinic or pyrrolic N atoms, also contribute to the activity, in accordance with previous findings. Indeed, the complexity of these materials does not allow us to unambiguously ascribe the catalytic activity to one specific surface site. For example, the preferential location of the N-dopants (edge vs in-plane, in the pores or outside the pores, etc.)^42,70^ or intrinsic carbon defects^43^ may also play a role. Furthermore, there is a certain spectral overlap between different N-moieties in these N–C structures in the XPS spectra, which further complicates establishing precise structure–property correlations.^67^

**Figure 6:**
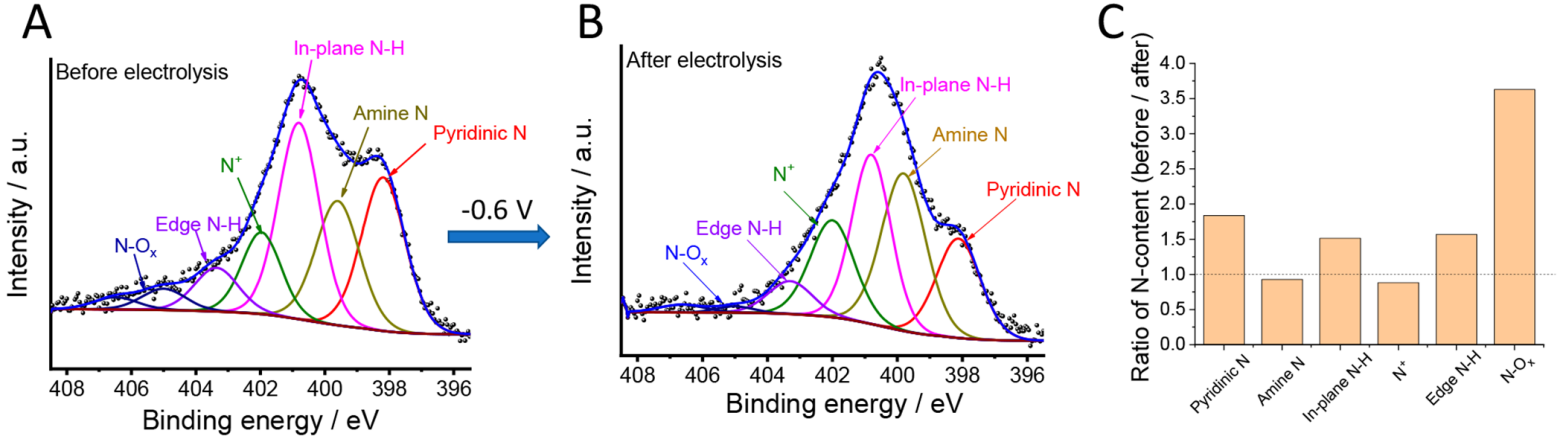
High-resolution N 1s spectra of a PoPD-C-NH_3_ electrode before (A) and after (B) a 2 h electrolysis in a CO_2_-saturated 0.1 M KHCO_3_ solution at −0.6 V. (C) Ratio of the amounts of different N-species in the PoPD-C-NH_3_ electrode before and after electrolysis.

To gain further insights into the reduction process, we recorded the XPS spectra of the best-performing PoPD-C-NH_3_ electrode before and after a 2 h electrolysis test. We observed a slow but steady decrease in the reduction current: *I*_CO_ dropped by 28%, while there was only a minor decrease in *I*_HER_ (Figure S16). This implies that the main active sites are different for HER and CO_2_R, which is also supported by the distinct trend in the *I*_CO_ and *I*_HER_ values presented above. In parallel with the decrease in CO formation over time, the relative N-content of the catalyst changed from 3.5 to 2.6 atom %. The fitting of the high-resolution N 1s spectra before and after the CO_2_R revealed even more interesting trends (Figure 6). While the amount of amine N atoms and N^+^ surface groups remained unchanged within experimental error, there was a drop in the pyridinic N—the N–H— and −NO_*x*_ contents. The greatest alteration was seen in the amount of −NO_*x*_ groups: a 72% decrease after electrolysis! This finding confirms our above assumption that the presence of −NO_*x*_ groups plays a major role in the CO_2_R process. Taking into account that *I*_CO_ declined only by 28%, while the amount of −NO_*x*_ groups decreased by 75%, we emphasize that the −NO_*x*_ groups are probably not the only active sites for CO_2_R but, on the basis of our findings, their presence seems to be necessary for the CO formation. There are several reports on the pyridinic N atoms being the active sites in similar materials.^27,28,45,71^ Our results also support the importance of these N-moieties as well as the N–H, as the relative amounts of these species also significantly decreased in the reaction.

### CO_2_ Adsorption/Desorption

The adsorption of reactants and intermediates plays a crucial role in determining the reactivity; therefore, we investigated whether the effect of the −NO_*x*_ groups is rooted in their favorable CO_2_ adsorption characteristics. We performed CO_2_ temperature-programmed desorption (TPD) and quartz crystal microbalance (QCM) investigations on the PoPD-C and PoPD-C-NH_3_ catalysts. These two samples were prepared from the same precursor and therefore only differed in their surface chemical compositions because of the NH_3_ activation step: namely, the higher −NO_*x*_ content.

**Figure 7:**
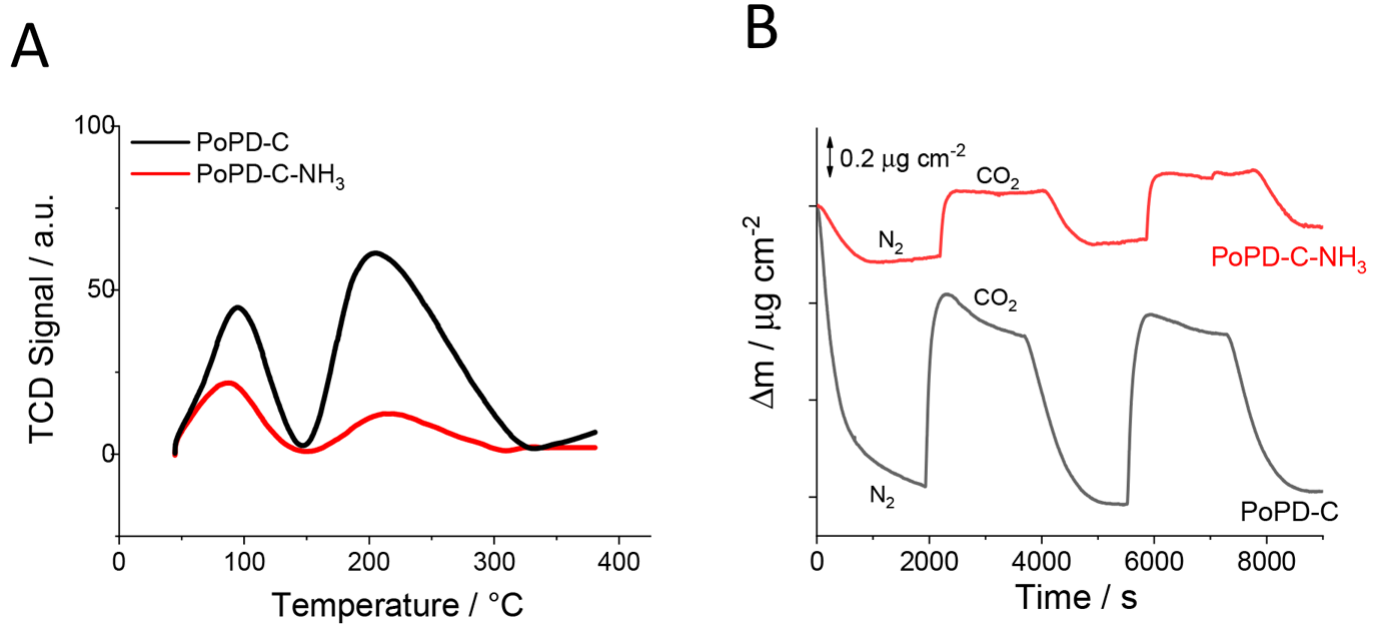
Temperature-programmed CO_2_ desorption profiles of PoPD-C and PoPD-C-NH_3_ powder samples (A). CO_2_ adsorption characteristics of PoPD-C and PoPD-C-NH_3_ thin layers studied by a quartz crystal microbalance (B).

The desorption of CO_2_ from the powder samples during the TPD analysis happened in two well-separated steps at around 90 and 210 °C, related to the weakly adsorbed and chemisorbed CO_2_, respectively. Somewhat surprisingly, both techniques revealed that the more active PoPD-C-NH_3_ adsorbs around 3 times *less* CO_2_ in comparison to the less active PoPD-C catalyst (Figure 7). The total desorbed CO_2_ was 0.02 mmol g^–1^ in the case of PoPD-C-NH_3_, while it was 0.07 mmol g^–1^ for PoPD-C. A similar trend was observed during the QCM measurements (at room temperature) on the thin-layer catalysts. The mass difference when the N_2_ atmosphere was changed to CO_2_ was 0.35 ± 0.03 μg cm^–2^ for PoPD-C-NH_3_ and 0.86 ± 0.06 μg cm^–2^ in the case of the PoPD-C catalyst.

The differences between PoPD-C-NH_3_ and PoPD-C come from the different total N-contents (6.5% vs 9.6%; Figure 2B). The ammonia treatment reduced the amount of overall basic sites (such as amine, −NH in plane and −NH edge, from 5% to 3%) where otherwise CO_2_ is most preferentially adsorbed. This implies a larger amount of CO_2_ trapping in the case of PoPD-C but not necessarily an improved TC or EC activity, as trapping CO_2_ can render CO_2_ inactive (i.e., for instance as spectator species as carbonates). In the TPD profile in Figure 7, it becomes clear that the low-temperature peak is much less affected than the high-temperature peak, which would be responsible for spectator species. Indeed, the fact that the increased −NO_*x*_ content of PoPD-C-NH_3_ did not result in enhanced CO_2_ adsorption suggests that the −NO_*x*_ sites are not preferential adsorption sites for CO_2_. Or at least the gain in CO_2_ adsorption resulting from the increased −NO_*x*_ content is lower than that we lose because of the decreased amount of other N-species. Unraveling the preferential CO_2_ adsorption sites, however, would require measurements able to give direct evidence (e.g., near-ambient-pressure XPS) on the process,^31^ which are out of the scope of this work.

### Theoretical Insights

**Figure 8:**
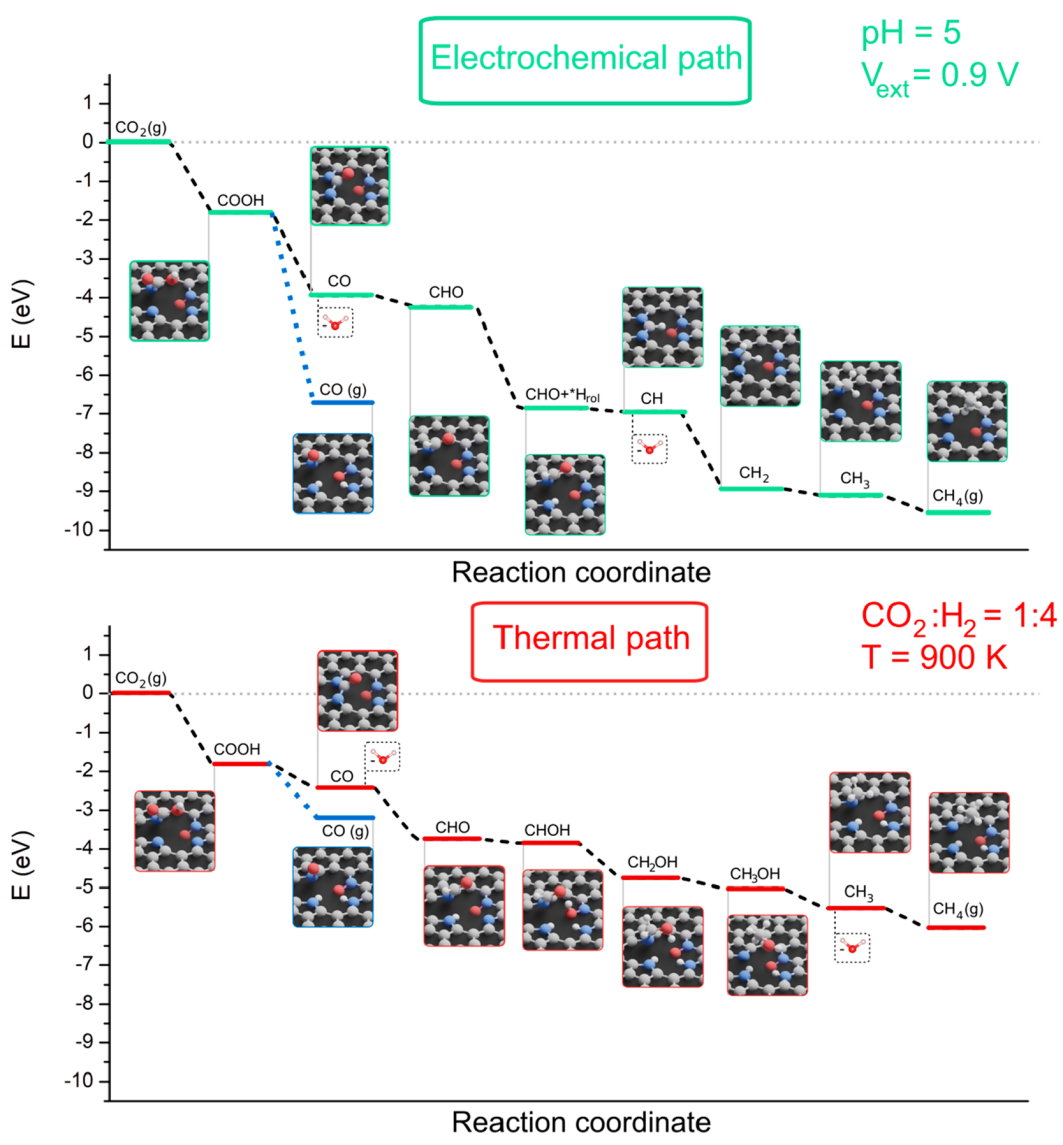
Energy profile of the electrochemical and thermal paths. Both have a pressure of 1 atm. The electrochemical path was done at 298.15 K. pH is not present on the thermal path, but it has a ratio of 1:4 for the injected CO_2_:H_2_. The last step to close the cycle, *H_din_ + *H_rol_ → H_2_(g) + 2*, is not shown.

Simulations for the TC and EC processes were performed by following standard gas-phase corrections for TC and the computational hydrogen electrode (CHE) model for EC to investigate multiple possible paths for producing CO and CH_4_ according to the literature.^72−75^ The optimum paths that worked for that system are summarized in Table 1 for CO and Table 2 for CH_4_ and in Figure 8. The details of the derivation of Gibbs energies for the TC and EC paths are presented in Tables S6–S18 in the Supporting Information, while the structures for the defects evaluated and the adsorption of the key intermediates are presented in Figures S17–S20.

**Table 1:**
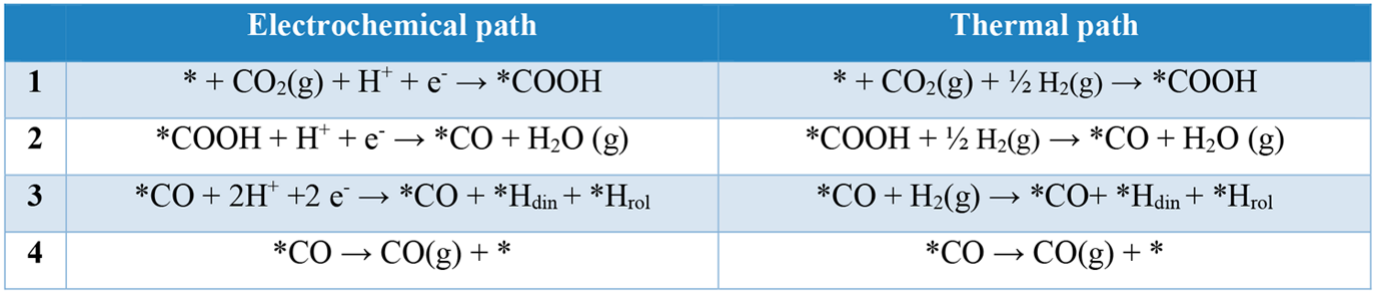
Comparison between the Electrochemical and Thermal Paths for CO Production^a^

a Asterisks represent the active sites where the fragment is bound to the surface. In the electrochemical path, H^+^ is provided by the solvent and e^–^ by the solid material at the applied potential. In the thermal path, there is no pH or applied potential and the required hydrogen comes from H_2_(g) or captured *H by the cavity. *H_rol_ denotes *H captured by pyrrolic N, and *H_din_ denotes *H captured by pyridinic N.

**Table 2:**
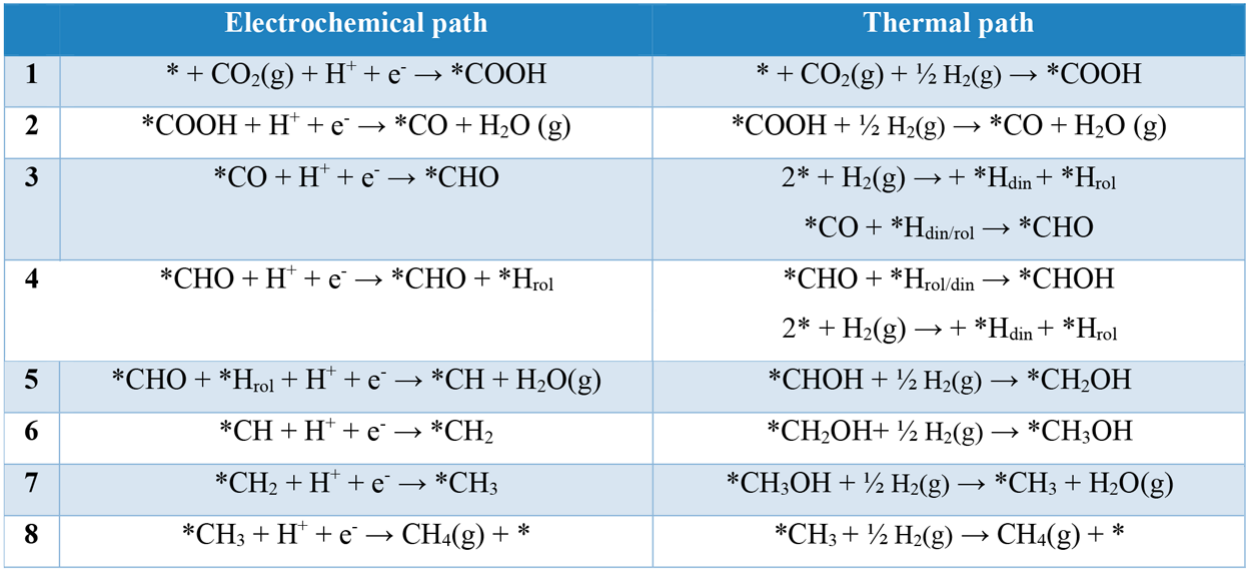
Comparison between the Electrochemical and Thermal Paths for CH_4_ Production^a^

a Asterisks represent the active sites where the fragment is bound to the surface. In the electrochemical path, H^+^ is provided by the solvent and e^–^ by the material at the applied voltage. In the thermal path, there is no pH or applied voltage and the required hydrogen comes from H_2_(g) or captured *H by the cavity. *H_rol_ denotes *H captured by pyrrolic N, and *H_din_ denotes *H captured by pyridinic N.

We observed that the surface with four N and one O (i.e., a cavity containing two pyrrolic, one pyridinic, and one pyridine N-oxide site) produces CO and CH_4_ under the experimental conditions of an external voltage of −0.9 V and pH > 5. This was the energetically most favorable geometric structure among the investigated structures in both the TC and EC reactions. This observation confirms the often-claimed importance of pyridinic N groups but puts their role into a different perspective. This mechanism also points to the important role of −NO_*x*_ moieties, as observed experimentally.

With the assumption of a constant volume and pressure, all Gibbs energies were computed using the standard formula
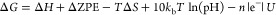
1where *H* is the enthalpy, ZPE the zero-point vibrational energy, *S* the entropy, *n* the number of electrons interchanged, and *U* the external applied voltage. Vibrational energies were accounted in the entropic (S) and enthalpy (H) terms. Translational and rotational terms have also been accounted for in the enthalpy. The thermal path was investigated at 900 K to simulate the 600 °C point and at 1000 K to simulate the 700 °C point. The main difference within the chemical equations is the replacement of H^+^ and e^–^ by *H or 1/2H_2_(g). This means that the required hydrogen comes either directly from the injected H_2_(g) or from the captured *H in the cavity. These *H species are on the pyrrolic N or pyridinic N of the cavity or both. We will use the notation *H_rol_ if it comes from a pyrrolic group and *H_din_ if it comes from the pyridinic group. In this case, there is no pH or applied voltage and the Gibbs energy can be rewritten as
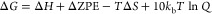
2where *Q* is the chemical quotient of the reaction. Under these conditions, the reactions are very similar between the EC and TC paths for CO formation as CO(g) is released when both pyrrolic and pyridinic sites are occupied by H. For CH_4_, the first two steps are the same as for the CO path. However, the third step is peculiar in the thermal path, as it first requires that both pyridinic and pyrrolic sites be occupied in the cavity for the reaction to occur. Then either a *H_rol_ or a *H_din_ species can be transferred. The difference in energy is less than 0.1 eV between the two possibilities, making them indistinguishable in Figure 8. In practice the transferring *H_din_ and leaving *H_rol_ in the cavity has the lowest energy. In step 4, we would have expected CHOH to be formed but we systematically observed that the extra hydrogen would go on the pyrrolic site, forming a *H_rol_ species. The thermal path on the fourth step used a *H_rol_ species if step 3 used a *H_din_ species or vice versa, an *H_din_ species if step 3 used a *H_rol_ species. To keep CHOH stable, both pyridinic and pyrrolic sites have to be occupied in the cavity in the thermal path.

The next steps require that the cavity be filled with both *H_rol_ and *H_din_ to work and high temperatures are used (i.e., would not work at 298.15 K). The capture of H_2_ by the cavity is more favorable with higher temperature. Step 5 in the electrochemical path releases a water molecule to form *CH, and then grows it until CH_4_ is formed in the last step, while the thermal path continues to grow *CHOH into *CH_2_OH up to *CH_3_OH and then only releases a water molecule to give *CH_3_ and finally CH_4_. This difference in the thermal and electrochemical paths may explain the experimentally observed ca. 1 magnitude difference in the CO:CH_4_ ratio in the two cases. The proposed thermal path works at 900 K and even better at 1000 K but not at 298.15 K, as the capture of the *H_rol_ and *H_din_ species is more difficult at room temperature (see Tables S14–S16). It should be noted that the final step 8 keeps the *H_rol_ and *H_din_ species on the surface.

Finally, we have identified possible deactivation routes involving the −NO_*x*_ active sites. The different stabilities found for the catalysts in Figure 4C and Figure S13 in the production of CO or CH_4_ comes from the fact that the N–O bond can be cleaved, which is preferentially happening for mechanisms where CO stays bound to the surface for a long time as required for the CH_4_ mechanism. Other deactivation mechanisms are presented in Figure S20. Those include −NO reduction through the H atoms stored in the surrounding of the defects. This route leads to water that can be eliminated and thus would be more effective under TC conditions than for the EC route due to both the thermal and entropic gain of the generated water molecule. The alternative deactivation route is bicarbonate formation, which is expected to be more likely under electrochemical conditions and could block the cavities in the carbon scaffold.

## Conclusions and Outlook

We revealed analogies between the TC and EC CO_2_ conversions, by investigating the same set of N–C catalysts in the two processes. The catalysts were synthesized by a sacrificial support method using different precursor polymers. This synthetic strategy resulted in similar pore structures of the catalysts, as confirmed by electron microscopic imaging and pore structure analysis using N_2_ adsorption/desorption. In contrast, the surface chemical composition, which was studied by XPS, varied between the samples originating from the different precursors. We studied the role of different surface functional groups in the reactions and found that the activity trends in the CO_2_R among the samples were very similar, independent of being thermally or electrochemically activated. We observed a higher CO-formation rate for the samples that were rich in −NO_*x*_ surface groups. The decrease in the CO_2_R activity over time was correlated with the loss of these moieties. DFT calculations revealed that an −NO_*x*_ group (pyridine N-oxide) needs to be in the proximity of two pyrrolic nitrogens and one pyridinic nitrogen for the CO_2_ conversion to take place. This work, therefore, highlights the importance of the −NO_*x*_ moieties (in addition to the pyridinic and pyrrolic N atoms in CO_2_ reduction), which were generally neglected in previous studies because of their relatively lower concentration in comparison to other N-functional groups. The mechanisms of CO formation were found to be very similar for the TC and EC paths, while the mechanism was different in the case of the CH_4_ product. In both cases, however, the protonation of the pyrrolic and/or pyridinic nitrogen atoms plays an important role. The revealed degradation mechanism (involving the −NO_*x*_ groups), together with the experimentally observed performance decrease, points toward further challenges of this catalyst family. The similarity found between the electrochemical CO_2_R and the thermal hydrogenation of CO_2_, however, may initiate knowledge transfer between the two disciplines. This could accelerate catalyst development by directly taking over materials proved to be efficient in one research field.

## Supporting Information Available

Catalyst characterization, additional catalytic activity and structure–property correlation data, and theoretical and computational details (PDF)

## Notes

The computed data is stored at ioChem-BD, DOI: 10.19061/iochem-bd-1-199.
