# Supplementary material for: CO2 Conversion on N-Doped Carbon Catalysts via Thermo- and Electrocatalysis: Role of C–NOx Moieties
Source: ACS Catal. 2022 Aug 4;12(16):10127–40. doi: 10.1021/acscatal.2c01589 (PMC9397536; doi:10.1021/acscatal.2c01589)
Supplement: Supplementary file 1 — cs2c01589_si_001.pdf [file cs2c01589_si_001.pdf]

Supporting Information

CO_2 Conversion on N-doped Carbon Catalysts via Thermal- and Electrocatalysis: the Role of C-NO_x Moieties

Dorottya Hursán,[1,3] Marietta Ábel[2,3], Kornélia Baán[2,3], Edvin Fako[4], Gergely F. Samu[1,3], Huu Chuong Nguyën[4], Núria López[4], Plamen Atanassov[5,6], Zoltán Kónya[2,3], András Sápi[2], Csaba Janáky*[,1,3]

[1]Department of Physical Chemistry and Materials Science, University of Szeged, H-6720 Szeged, Hungary
[2]Department of Applied and Environmental Chemistry, University of Szeged, H-6720, Szeged, Hungary
[3]Interdisciplinary Excellence Centre, University of Szeged, H-6720 Szeged, Hungary
[4]Institute of Chemical Research of Catalonia, The Barcelona Institute of Science and Technology, 43007 Tarragona, Spain
[5]Department of Chemical and Biomolecular Engineering, University of California Irvine, 92697 Irvine, CA, USA
[5]National Fuel Cell Research Center, University of California Irvine, 92697 Irvine, CA, USA

*corresponding authors email: janaky@chem.u-szeged.hu (C. Janáky)

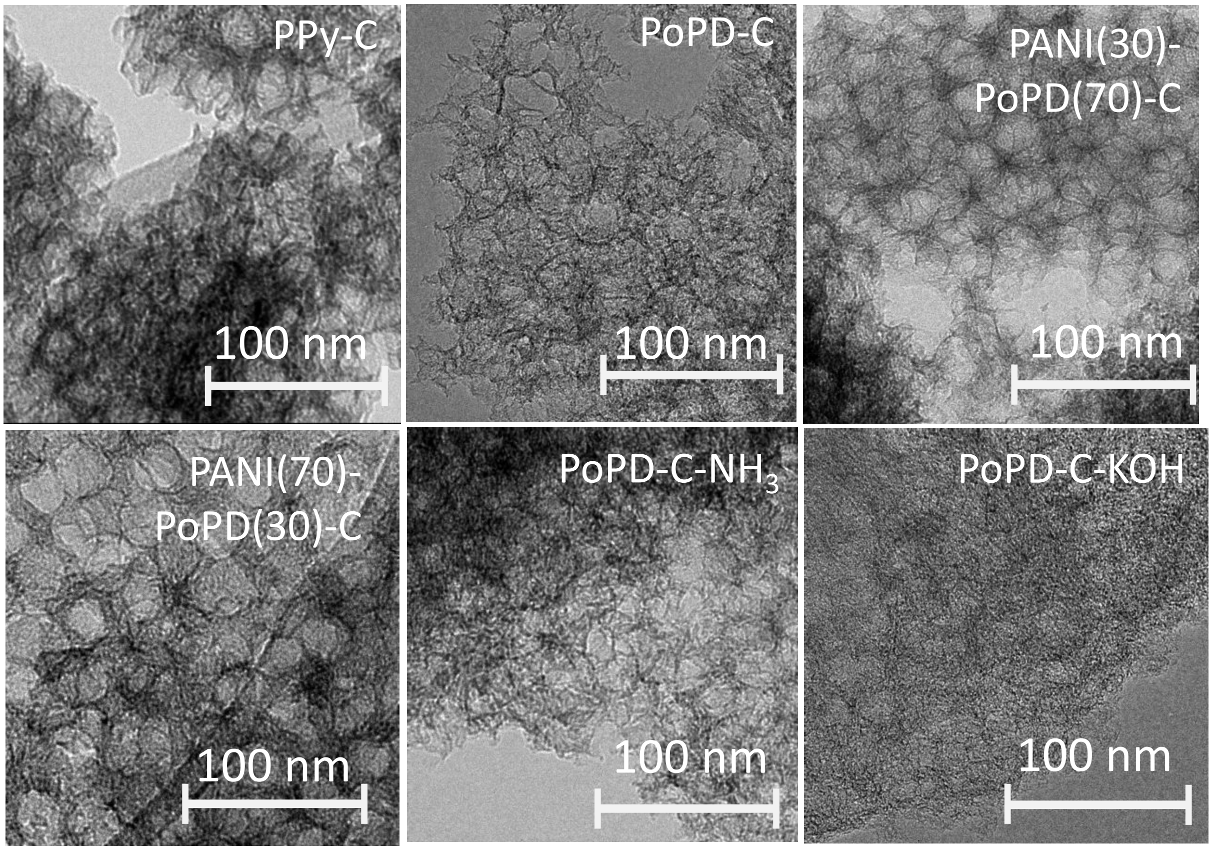

Figure S1. TEM images of PPy-C, PoPD-C, PANI(30)-PoPD(70)-C, PANI(70)-PoPD(30)-C, PoPD-C-NH_3 and PoPD-C-KOH.

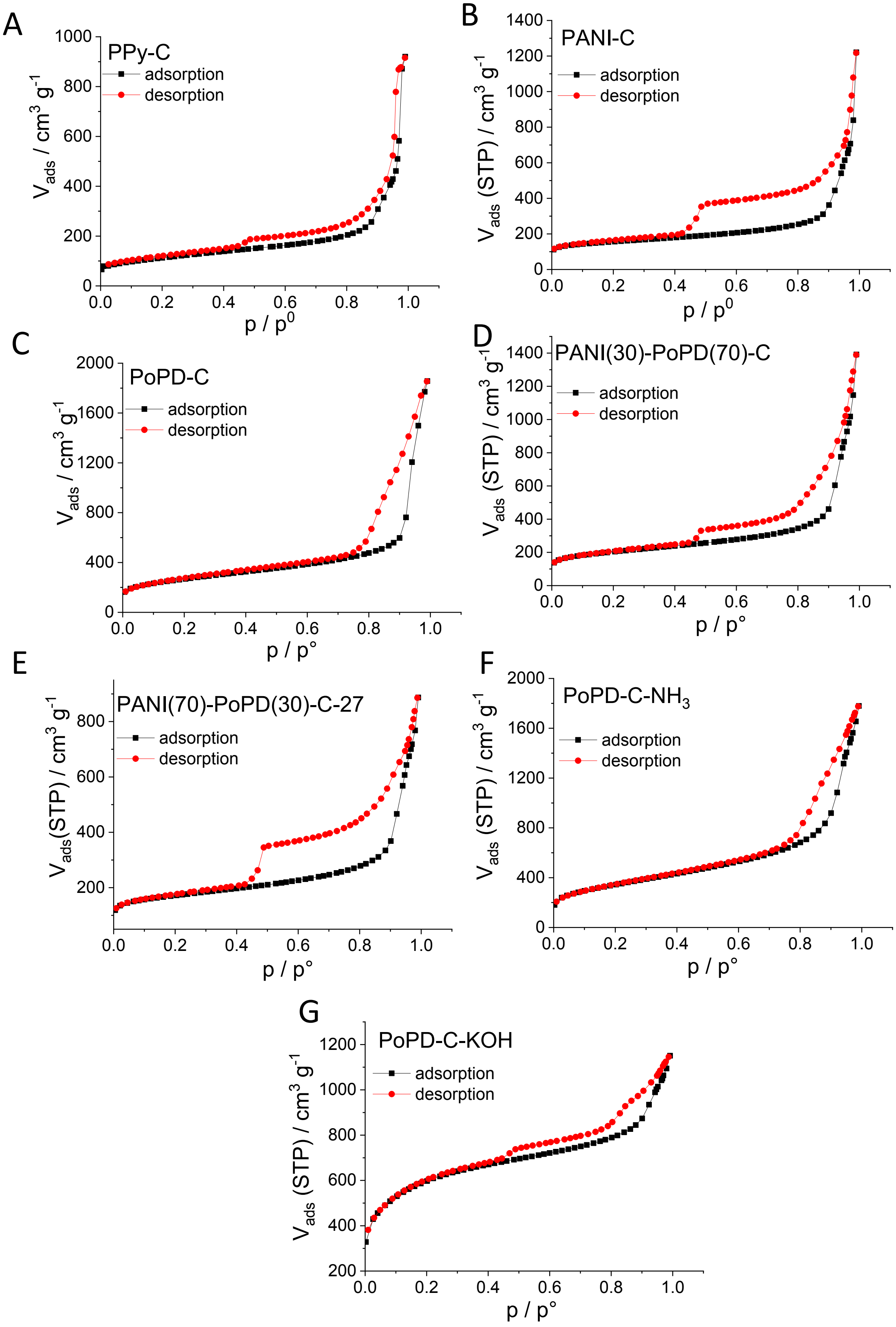

Figure S2. N_2 adsorption / desorption isotherms of the catalysts.

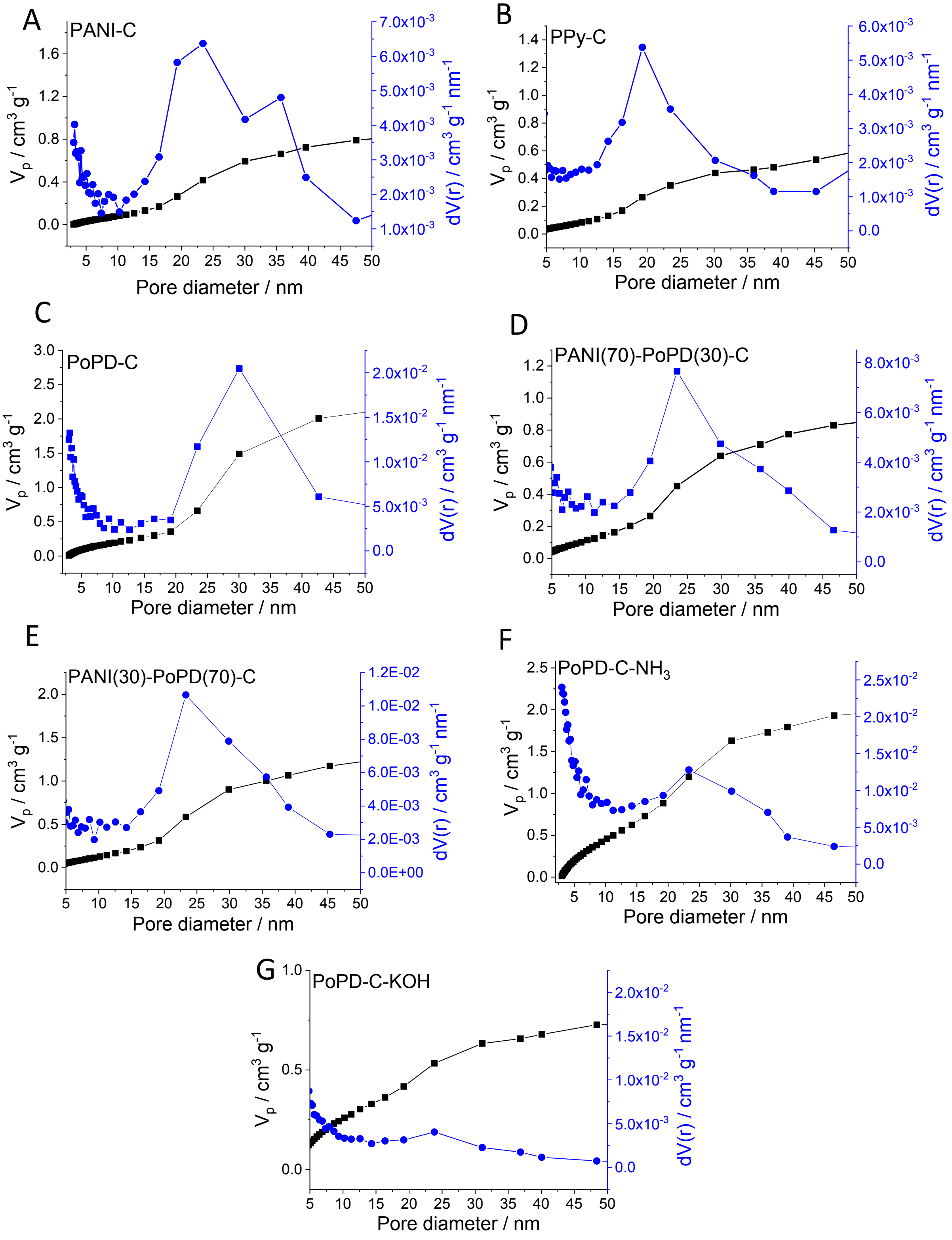

Figure S3. Pore-size distribution curves of PANI-C (A), PPy-C (B), PANI(30)-PoPD(70)-C (C), PANI(70)-PoPD(30)-C (D) PoPD-C-NH_3 (E) and PoPD-C-KOH (F) in the mesopore range, calculated by the BJH-method.

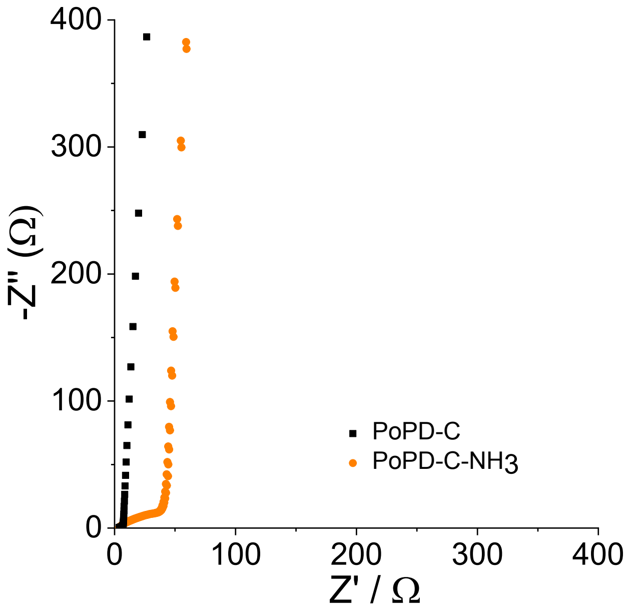

Figure S4. Electrochemical impedance spectra of the PoPD-C and PoPD-C-NH_3 electrodes in an Ar-purged 1 M Na_2SO_4 solution.

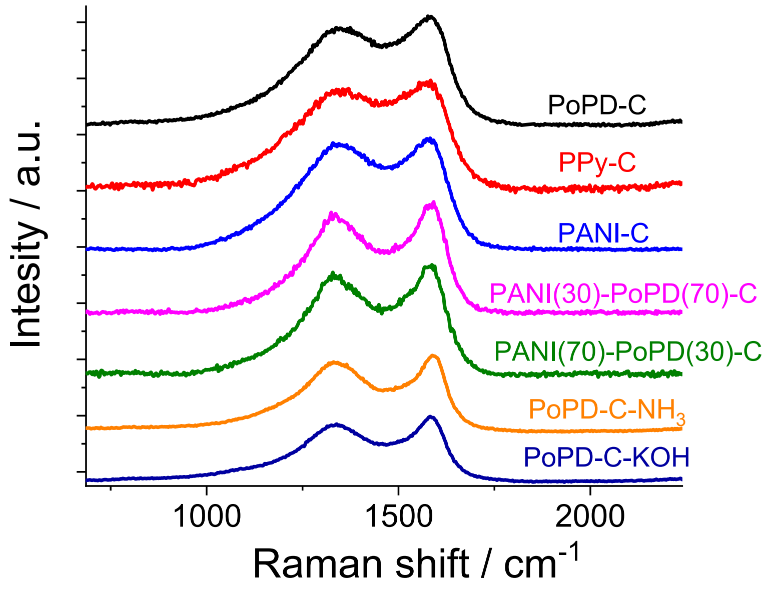

Figure S5. Raman spectra of the studied catalysts.

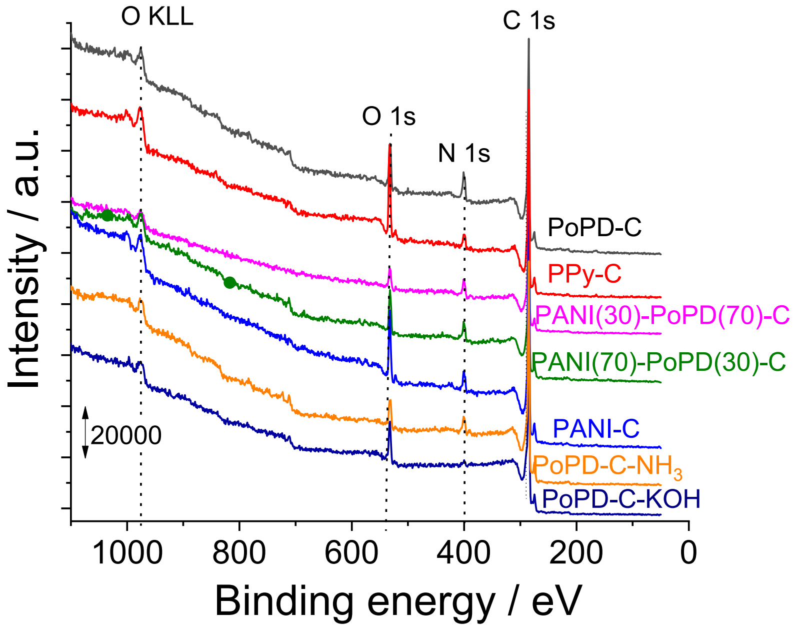

Figure S6. Survey XPS spectra of the studied catalysts.

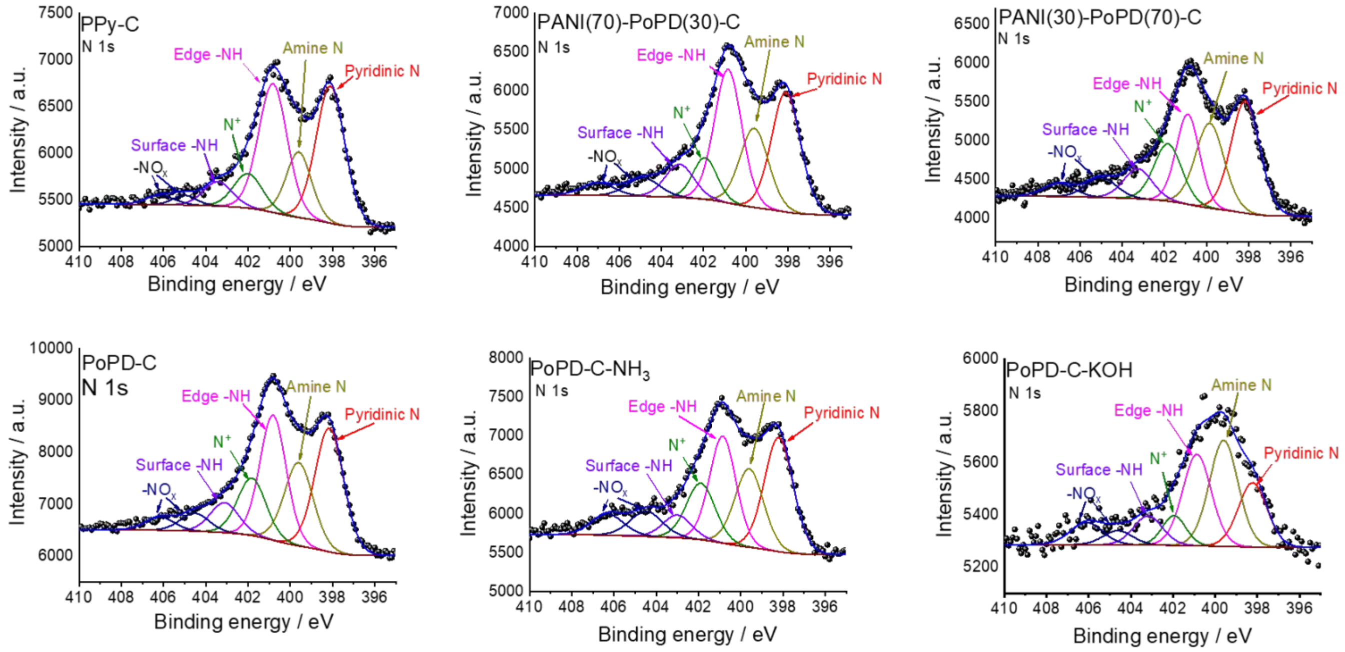

Figure S7. Fitting of the N1s region of the XPS spectra of the studied catalysts.

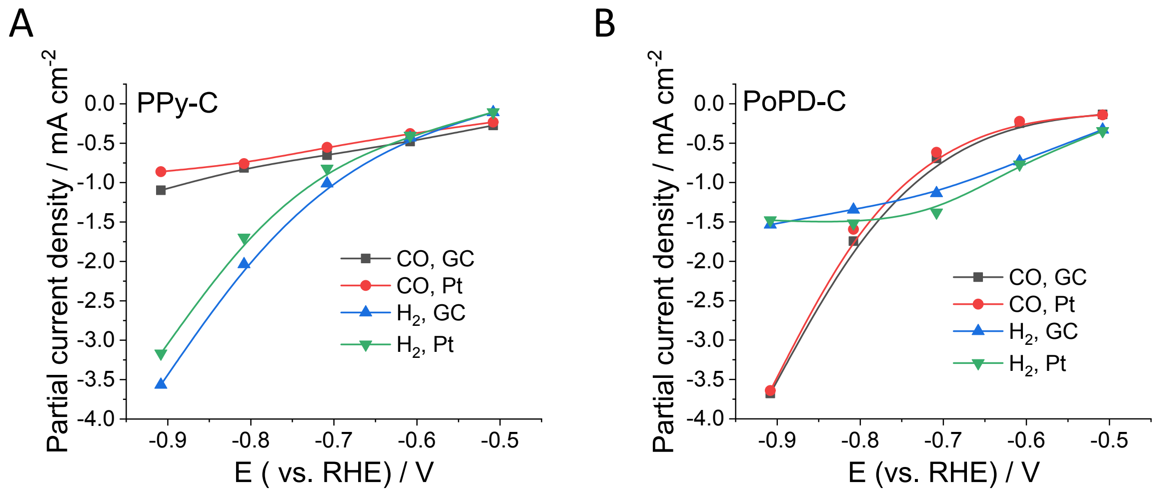

Figure S8: .Partial current densities of CO and H_2 revorded on PPy-C (A) and PoPD-C (B) electrodes, measured with Pt and glassy carbon (CG) counterelectrodes. Measurements were performed in a CO_2-saturated 0.1 M $KHCO_3$-solution.

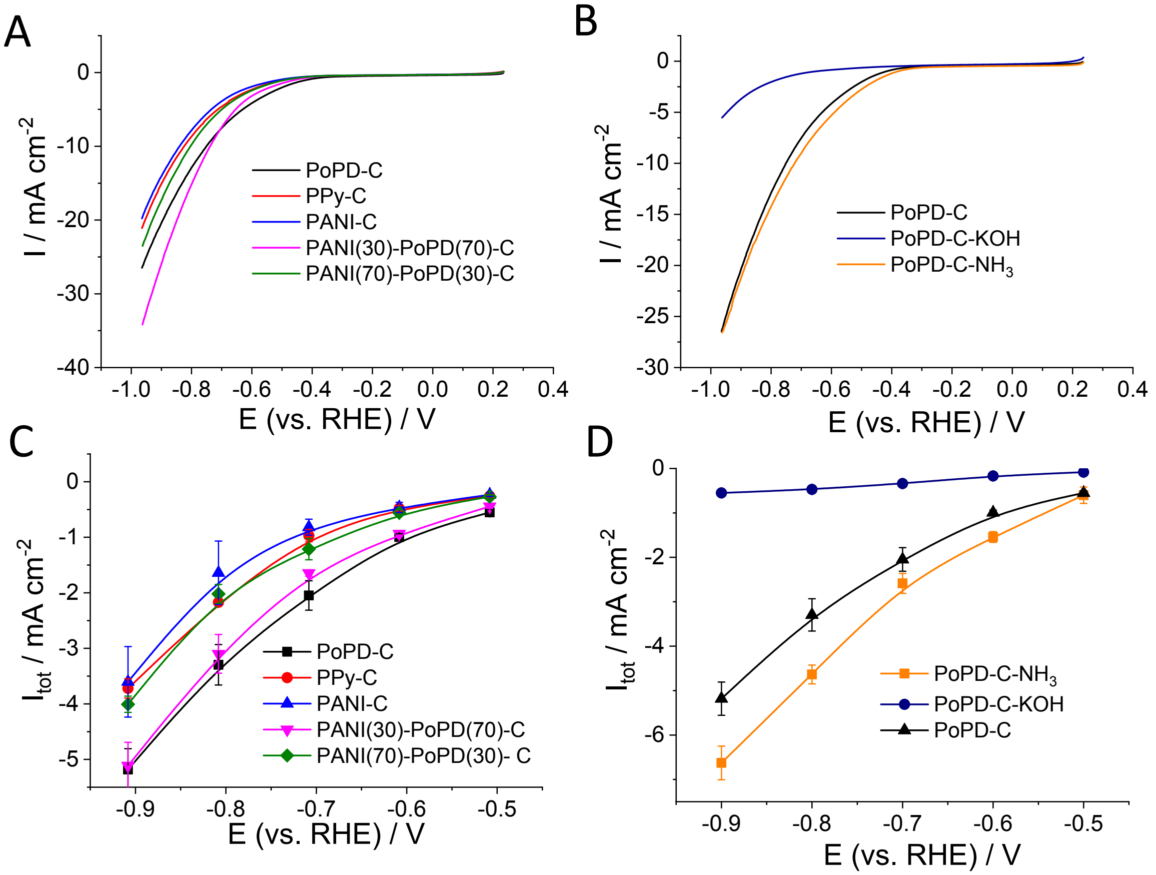

Figure S9. (A,B): LSV curves of the studied catalysts, recorded in a CO_2-saturated 0.5 M $KHCO_3$ solution with 5 mV s^{-1} sweep rate. (C,D): Total current densities measured in the 40th minute of the potentiostatic electrolysis in a CO_2-saturated 0.1 M $KHCO_3$ solution. Lines serve only as a guide for the eye.

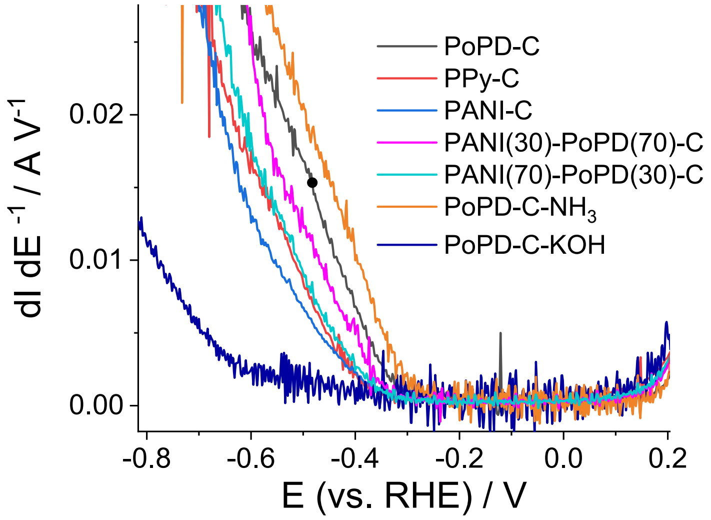

Figure S10. First derivatives of the LSV curves in Fig.S9. to determine the onset potentials of the reduction reaction.

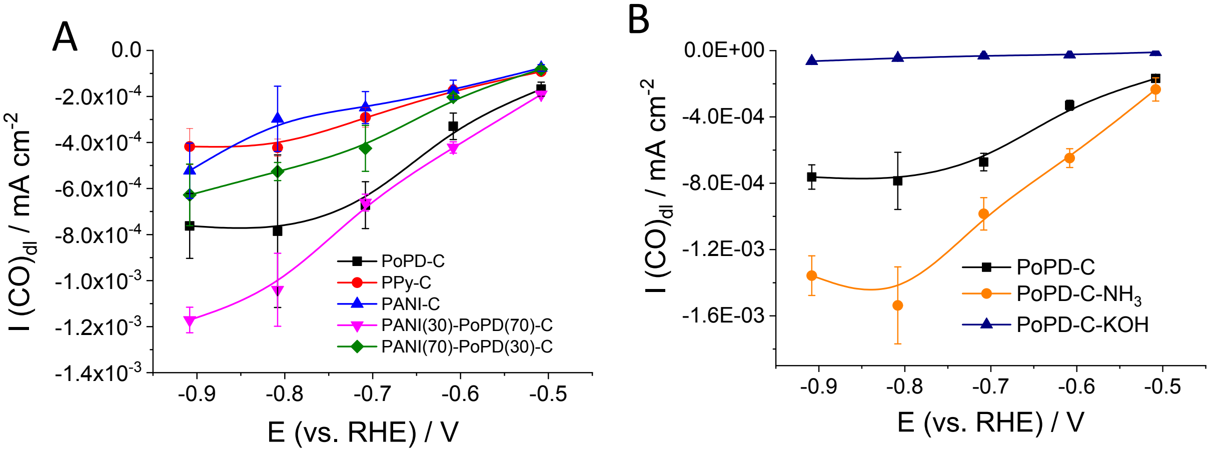

Figure S11. CO partial current densities on the studied N-C catalysts, normalized by the roughness factor of the electrodes (1.00 mg cm^{-2} loadings). Measurements were performed in a CO_2-saturated $KHCO_3$ solution. Lines serve as a guide for the eye.

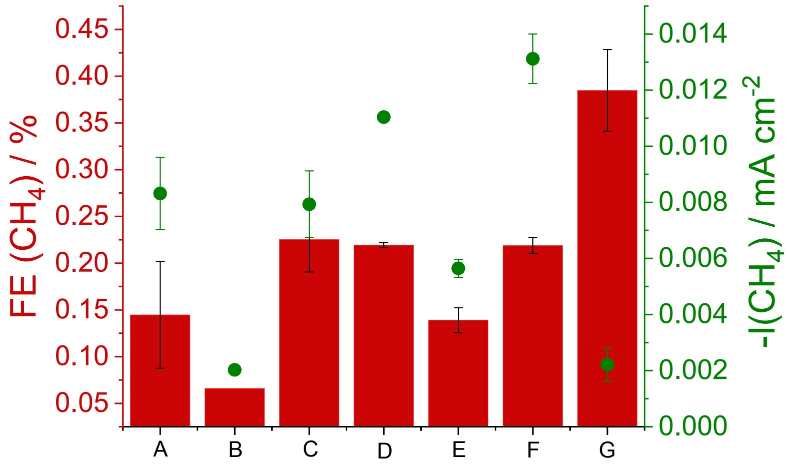

Figure S12. Faradaic efficiencies and partial current densities of methane on the studied N-C catalysts. A: PoPD-C; B: PPy-C; C: PANI-C; D: PANI(30)-PoPD(70)-C; E: PANI(70)-PoPD(30)-C; F:PoPD-C-NH_3; G: PoPD-C-KOH.

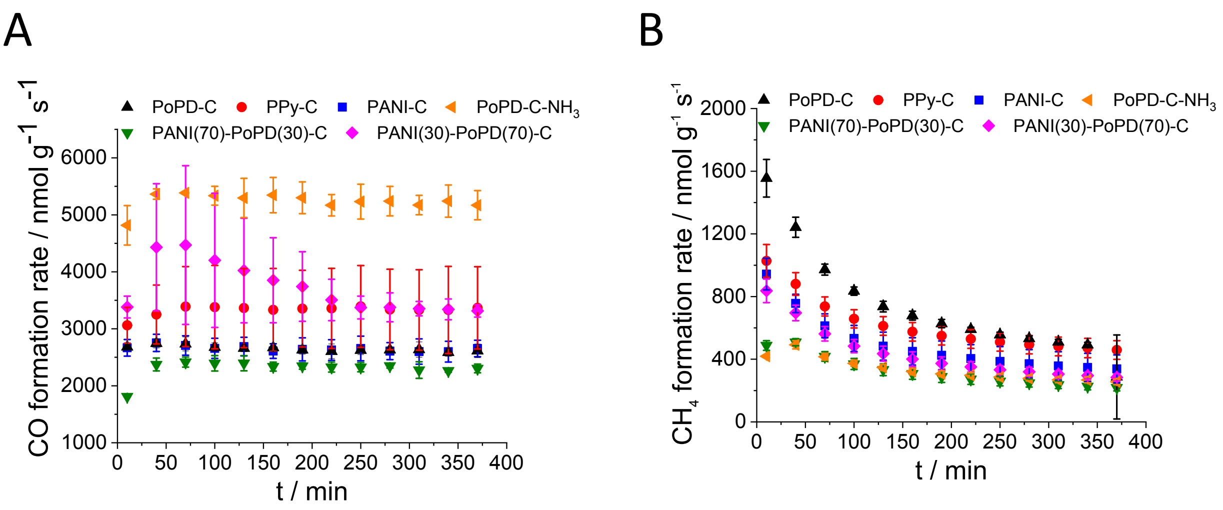

Figure S13. Stability of the TC CO_2 conversion process on the studied catalysts. (A): CO formation rate, (B): CH_4 formation rate.

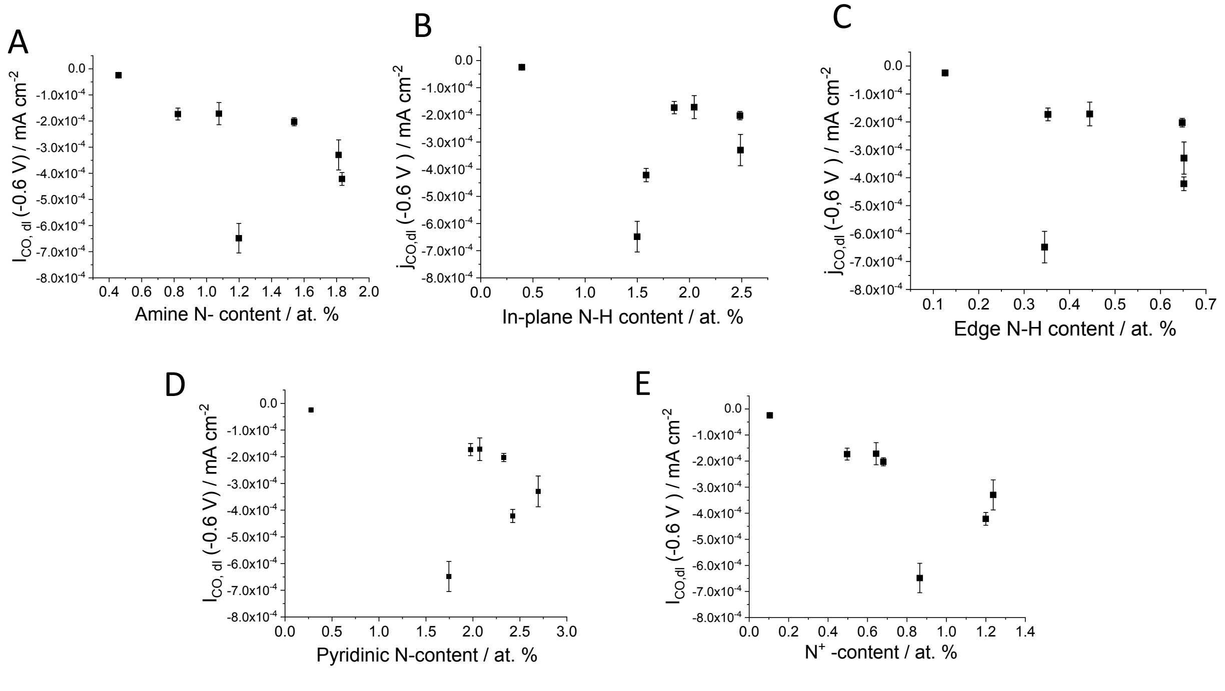

Figure S14. Correlation between the amount of different N-species and the EC CO_2 reduction activity (CO partial current densities normalized by the double layer capacitance value) of the studied catalysts.

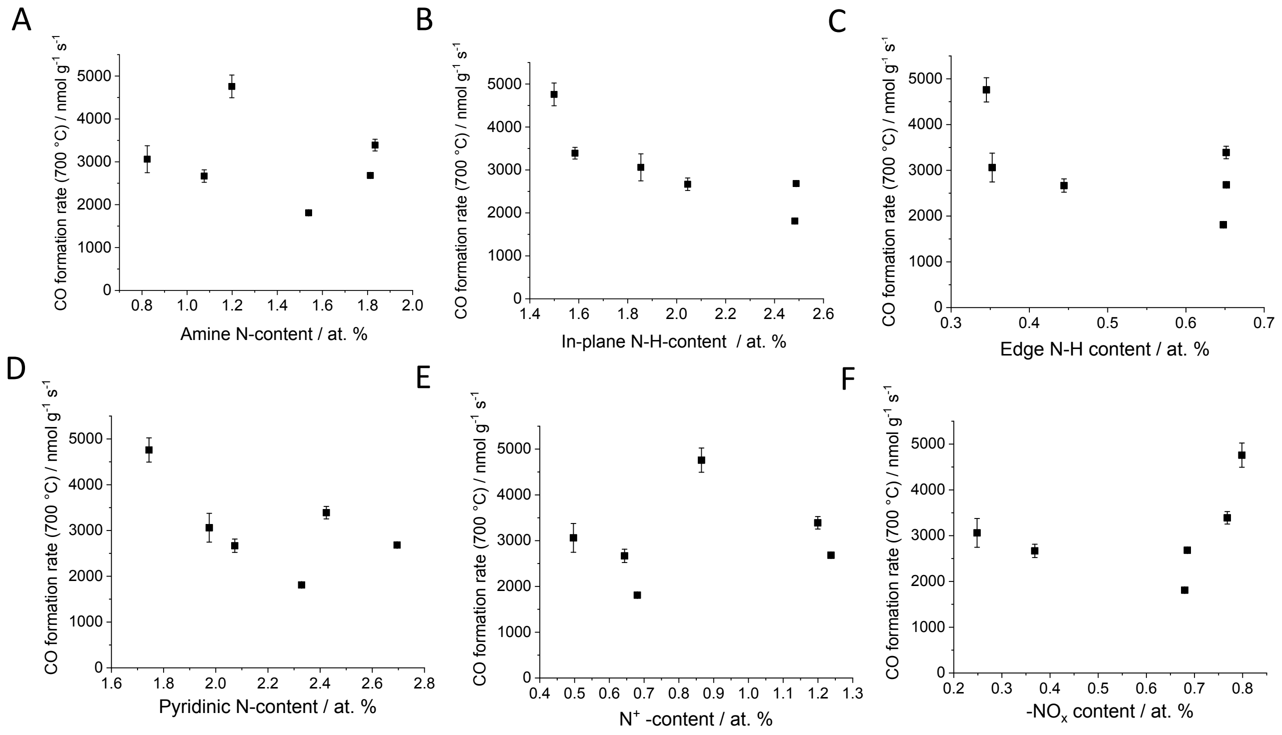

Figure S15. Correlation between the amount of different N-species and the TC CO formation rate.

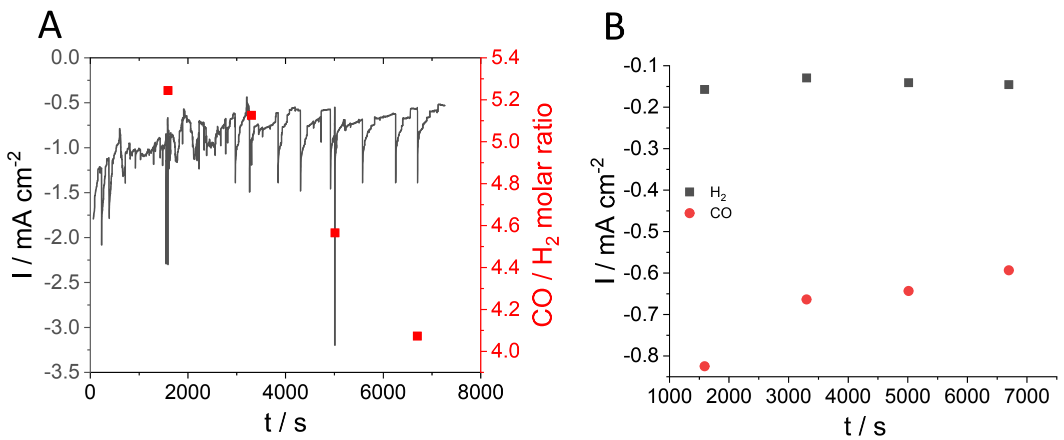

Figure S16. Potentiostatic electrolysis on a PoPD-C-NH_3 electrode in a CO_2-saturated 0.1 M $KHCO_3$-electrolyte at -0.6 V (vs. RHE). XPS analysis of the electrode was performed before and after electrolysis. (A): Chronoamperometric curve and the molar ratio of the formed CO and H_2. (B): Partial current densities of H_2 and CO during electrolysis.

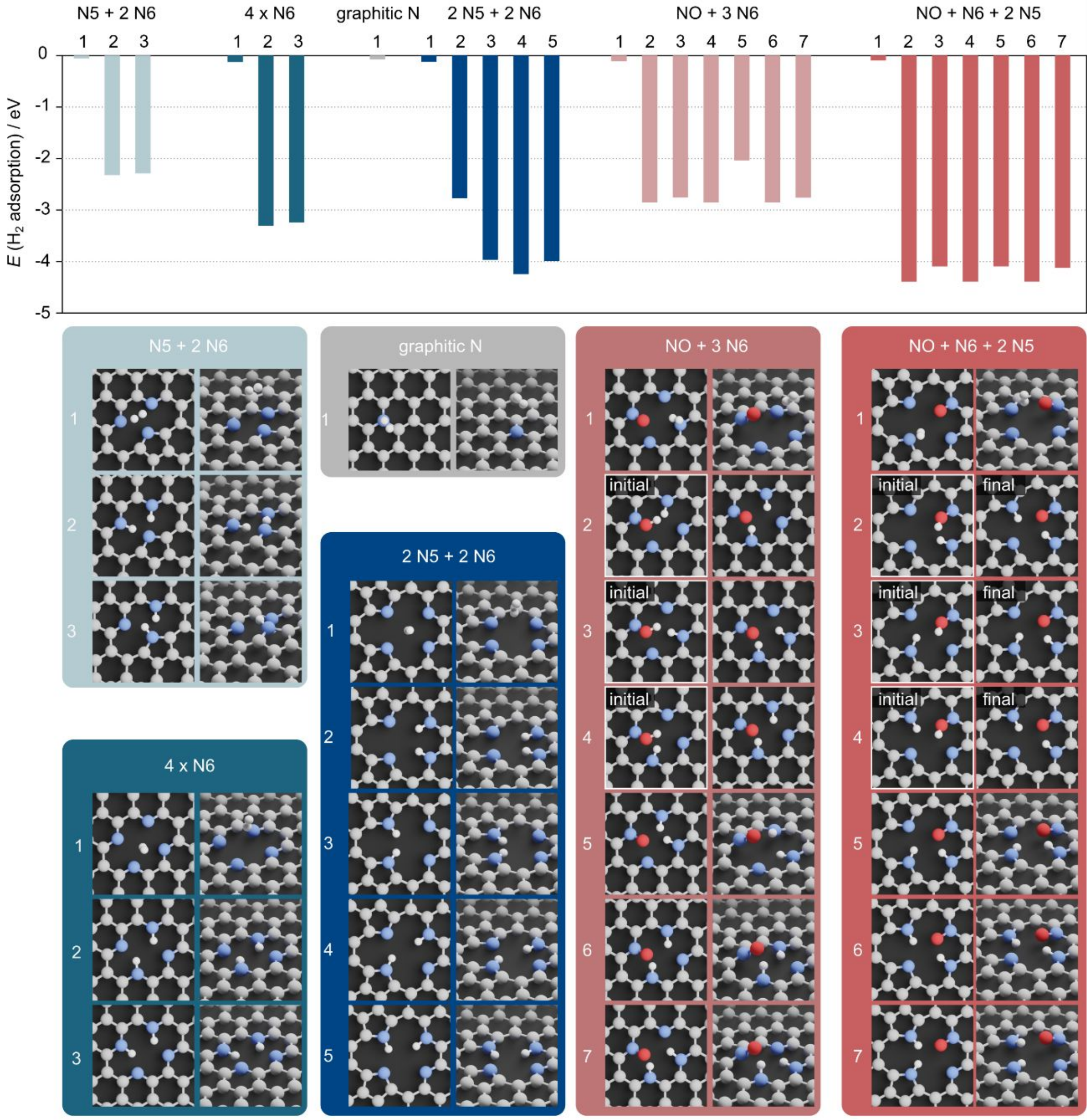

Figure S17. Hydrogen adsorption over defects containing graphitic N, pyrrolic (N5), pyridinic (N6) and oxo-pyridinic (NO) Nitrogen atoms. Relaxed structures corresponding to hydrogen physisorption (1), as well as activated H adsorption (2-7) are shown in the bottom panel. The energy plots are derived from the electronic energy of the empty defects and isolated H_2 molecules, versus configurations shown in the bottom inset.

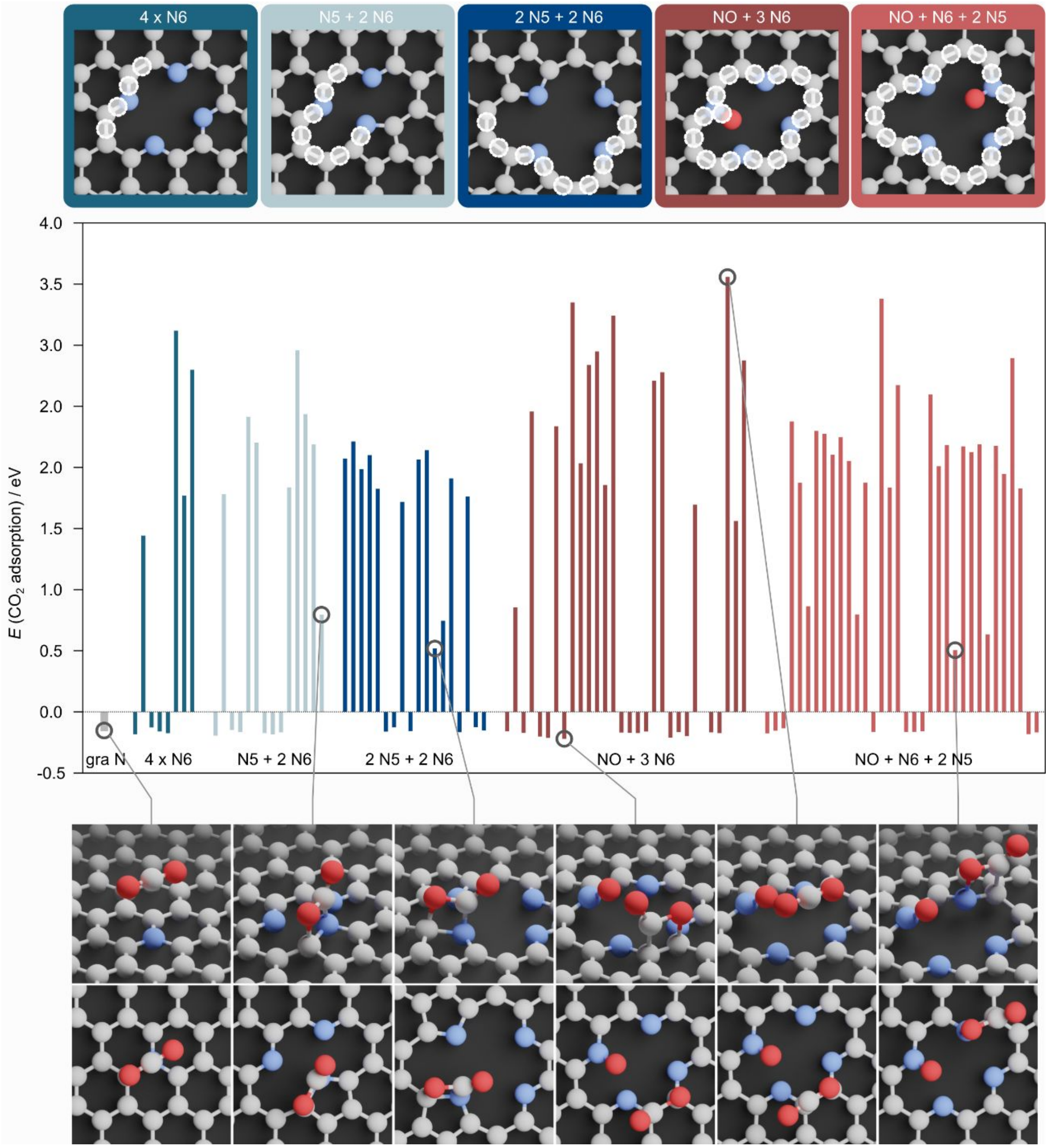

Figure S18. Electronic adsorption energy of CO_2 at the defect edges. CO_2 adsorption was computed over all nonsymetrically identical bridge sites of the corresponding defects (top inset). CO_2 adsorption was found to happen in two modes (i) vdW adsorption with adsorption energies of around 0.2 eV and (ii) Configurations in which 4-member rings are formed leading to typically highly endothermic adsorption energies in the 0.5 - 3.5 eV range depending on the defect.

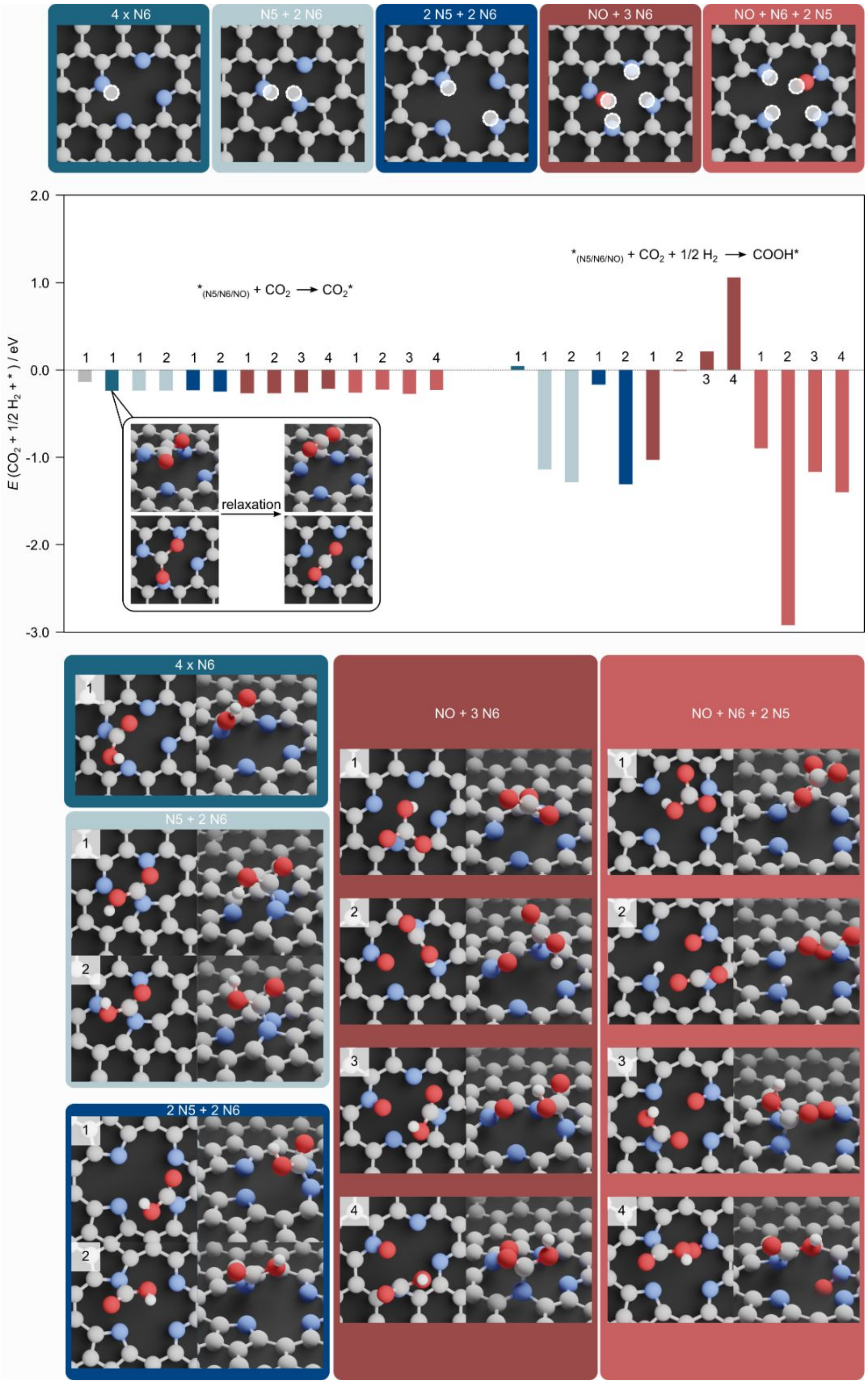

Figure S19. Electronic adsorption energies of CO_2 (left) and "one step" CO_2+1/2H_2 adsorption (right) over all symmetrically non-equivalent sites of the examined defects (top inset). No local minima were found corresponding to chemisorbed CO_2 molecules bound via a direct C(CO_2)-N(defect) bond (left), resulting in vdW adsorbed configurations. However, stable COOH motifs were found for several defect motifs (motifs), most notably on the pyrrolic N atoms (as in the NO+N6+2N5 defect).

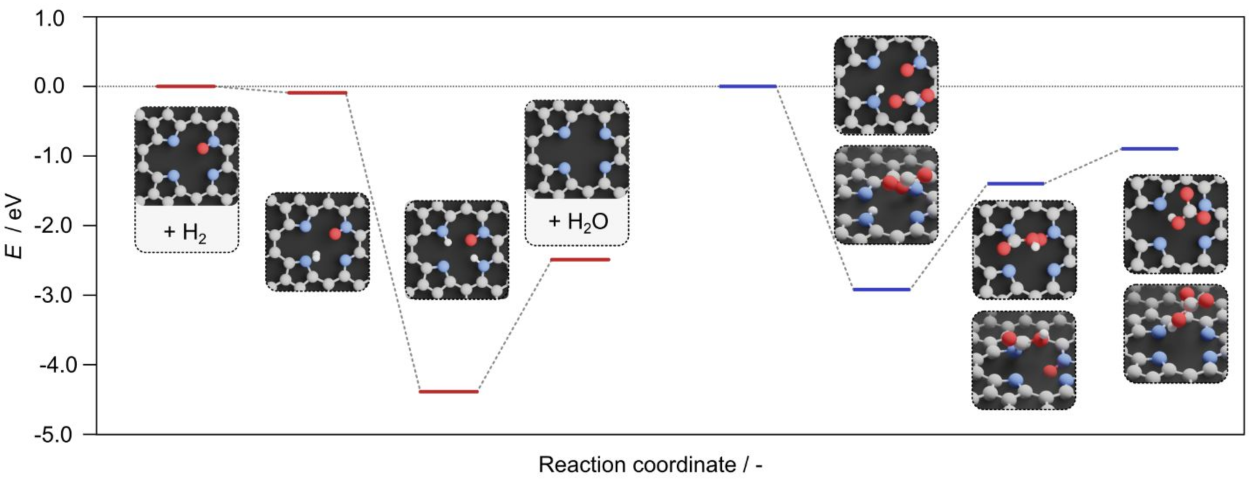

Figure S20. Deactivation pathways (electronic energy, referenced to isolated ½ H_2 and CO_2) of the oxo-pyridinic N containing defects, that indicate the possible deactivation via *NO reduction (red), and bicarbonate formation (blue).

Catalyst	Roughness factor (CV)	BET surface area / $m^2\ g^{-1}$
**PoPD-C**	2293	930
**PPy-C**	1642	403
**PANI-C**	1794	563
**PANI(30)-PoPD(70)-C**	1825	727
**PANI(70)-PoPD(30)-C**	1791	621
**PoPD-C- NH_3**	1982	1233
**PoPD-C-KOH**	4060	2182

Table S1. Roughness factors determined from the double layer capacitance values (cyclic voltammetry) and BET surface areas calculated from the N2 asorption / desorption isotherms of the studies N-C samples.

Catalyst	E_{onset} / V (vs. RHE)	Activation energy / kcal mol^{-1}
**PoPD-C**	-0.318	8.77
**PPy-C**	-0.356	10.48
**PANI-C**	-0.352	11.05
**PANI(30)-PoPD(70)**	-0.337	7.07
**PANI(70)-PoPD(30)**	-0.344	11.18
**PoPD-C-NH3**	-0.297	10.63
**PoPD-C-KOH**	-0.616	N/A

Table S2. Onset potentials of the EC reduction reaction for the different samples, determined from the derivatives of the LSV curves. Activation energy of the TC CO-formation.

Catalyst	Rel. %		
	C	N	O
**PoPD-C**	82.86	9.57	6.46
**PPy-C**	80.24	5.75	13.52
**PANI-C**	83.90	6.65	8.98
**PANI(30)-PoPD(70)-C**	87.13	8.46	4.41
**PANI(70)-PoPD(30)-C**	84.25	8.36	7.39
**PoPD-C-NH_3**	89.13	6.45	4.42
**PoPD-C-KOH**	91.70	1.53	6.78

Table S3. Elemental composition of the studied catalysts, determined from XPS measurements.

Catalyst	Rel. %					
	Pyridinic N	Amine N	In plane N-H	N^+	Edge N-H	$N-O_x$
PoPD-C	28.16	18.94	18.94	12.93	6.81	7.16
PPy-C	34.35	14.32	14.32	8.64	6.13	4.32
PANI-C	31.17	16.18	16.18	9.68	6.68	5.54
PANI(30)-PoPD(70)-C	28.65	21.67	21.67	14.18	7.70	9.08
PANI(70)-PoPD(30)-C	27.86	18.41	18.41	8.14	7.75	8.13
PoPD-C-NH_3	27.04	18.58	18.58	13.41	5.35	12.38
PoPD-C-KOH	18.12	29.91	29.91	6.84	8.24	11.16

Table S4. Relative amounts of the different N-species in the studied catalysts determined from XPS measurements.

Catalyst	I_D / I_G
PoPD-C	0.88 ± 0.03
PPy-C	0.92 ± 0.01
PANI-C	0.95 ± 0.01
PANI(30)-PoPD(70)-C	0.91 ± 0.03
PANI(70)-PoPD(30)-C	0.97 ± 0.01
PoPD-C-NH_3	0.87 ± 0.02
PoPD-C-KOH	0.87 ± 0.00

Table S5. Intensity ratios of the D and G bands in the Raman spectra of the studied catalysts.

Theoretical and computational details

For the calculation of the Gibbs energies for the thermal path, we have used

$$\Delta G = \Delta H + \Delta ZPE - T\,\Delta S + 10\, k_b T\ \ln(Q)$$

While the pH is not present in the thermal path, the term 10 k_bT ln(Q) has been estimated for each reaction with

$Q=[C_{product}]/([C_{reactant}][{*}H])$

Except in the first step, there are not extra C atoms playing in the reaction, and we have assumed $[C_{product}] \approx [C_{reactant}] \approx [CO_2]$.

Assuming to loss of hydrogen $[{*}H] \approx 2[H_2(g)]$, therefore

$Q \approx [CO_2]/([CO_2]\ 2[H_2(g)])$

Since the ratio of CO_2:H_2 is 1:4 we have $[H_2]=4[CO_2]$ thus

$Q \approx 1/(2{*}4) \approx 1/8$

The translational and rotational contribution to the enthalpy was approximated as 3/2 k_bT

The vibrational contribution of the entropy was approximated as

$$S_{vib} = R\sum_i\left[\frac{h\nu_i}{e^{h\nu_i/k_bT}-1} - ln\left(1-e^{-h\nu_i/k_bT}\right)\right]$$

Where R is the is ideal gas constant, h is the Planck constant, ν_i is the frequency, k_b is the Boltzmann constant. For all tables we named the surfaces with the convention ***clean*** is the basic 4N and a O cavity, ***hrol*** is the cavity with a pyrrolic H, ***hdin*** is the cavity with a H pyridinic and ***hh*** with both pyrrolic and pyridinc. All applied voltage are vs RHE. All numbers in the following tables are given in eV.

The following CO path without allowing *H transfer was investigated:

1. $* + CO_2(g) + H^+ + e^- \rightarrow {*}COOH$
2. $*COOH \rightarrow COO^- + {*}H$
3. $*COO^- + H^+ + e^- \rightarrow {*}CO + H_2O\ (g)$
4. $*CO \rightarrow CO\ (g)$

Surface	ΔG(*COOH) (eV)	ΔG(*CO) (eV)	ΔG(CO)(g) (eV)	Overpotential (eV)
**clean**	-1.809	-2.139	1.804	1.804
**hrol**	-1.219	0.330	-1.255	0.330
**hdin**	-1.771	-1.256	0.883	0.883
**hh**	-0.611	0.273	-1.806	0.273

Table S6. Gibbs energies for the electrochemical CO path without permitting H transfer from the cavity at 298.15K, V_{ext}=0.9 V, pH=5.

Surface	ΔG(*COOH) (eV)	ΔG(*CO) (eV)	ΔG(CO)(g) (eV)	Overpotential (eV)
**clean**	-0.962	-0.761	2.791	2.791
**hrol**	-0.372	1.709	-0.268	1.709
**hdin**	-0.923	0.122	1.869	1.869
**hh**	0.236	1.651	-0.819	1.651

Table S7. Gibbs energies for the thermal CO path without permitting H transfer from the cavity at 298.15K, V_{ext}=0, Q=1/8.

Surface	ΔG(*COOH) (eV)	ΔG(*CO) (eV)	ΔG(CO)(g) (eV)	Overpotential (eV)
**clean**	-1.812	-0.616	3.028	3.028
**hrol**	-1.242	1.777	0.064	1.777
**hdin**	-1.769	0.243	2.126	2.126
**hh**	-0.657	1.716	-0.460	1.716

Table S8. Gibbs energies for the thermal CO path without permitting H transfer from the cavity at 900K, V_{ext}=0, Q=1/8.

Surface	ΔG(*COOH) (eV)	ΔG(*CO) (eV)	ΔG(CO)(g) (eV)	Overpotential (eV)
**clean**	-2.001	-0.583	2.975	2.975
**hrol**	-1.434	1.799	0.011	1.799
**hdin**	-1.957	0.273	2.073	2.073
**hh**	-0.851	1.737	-0.387	1.737

Table S9. Gibbs energies for the thermal CO path without permitting H transfer from the cavity at 1000K, V_{ext}=0, Q=1/8.

The path without allowing H transfer from the cavities all have an overpotential > 0 and are therefore not thermodynamically meaningful. The optimal path presented in the paper follow the reaction presented in Table 1 of the manuscript. For the electrochemical path:

	Electrochemical path	Thermal path
**1**	$* + CO_2(g) + H^+ + e^- \rightarrow *COOH$	$* + CO_2(g) + ½ H_2(g) \rightarrow *COOH$
**2**	$*COOH + H^+ + e^- \rightarrow *CO + H_2O$ (g)	$*COOH + ½ H_2(g) \rightarrow *CO + H_2O$ (g)
**3**	$*CO + 2H^+ +2 e^- \rightarrow *CO + *H_{din} + *H_{rol}$	$*CO + H_2(g) \rightarrow *CO+ *H_{din} + *H_{rol}$
**4**	$*CO \rightarrow CO(g) + *$	$*CO \rightarrow CO(g) + *$

Table S10. Optimal CO path.

	ΔG(1) (eV)	ΔG(2) (eV)	ΔG(3)(g) (eV)	ΔG(4)(g) (eV)
**298K,pH=5,V=0.9**	-1.809	-2.139	-0.934	-1.806
**298K,Q=1/8**	-0.962	-0.761	-0.106	-0.819
**900K,Q=1/8**	-1.812	-0.616	-0.222	-0.460
**1000K, Q=1/8**	-2.001	-0.583	-0.254	-0.387

Table S11. Gibbs energy for the optimal CO path.

Electrochemical CH_4 paths without *H transfer from the cavity has been investigated.

1. $* + CO_2(g) + H^+ + e^- \rightarrow *HCOO$
2. $*HCOO + H^+ + e^- \rightarrow *CO + H_2O$ (g)
3. $*CO + H^+ + e^- \rightarrow *CHO$
4. $*CHO + H^+ + e^- \rightarrow *CHOH$
5. $*CHOH + H^+ + e^- \rightarrow *CH + H_2O(g)$
6. $*CH + H^+ + e^- \rightarrow *CH_2$
7. $*CH_2 + H^+ + e^- \rightarrow *CH_3$
8. $*CH_3 + H^+ + e^- \rightarrow CH_4(g) + *$

It turns out however that step 4 cannot produce *CHOH but always converge to a $*H_{rol}$ on the clean surface.

	1	2	3	4	5	6	7	8	
*Surface	ΔG(HCOO)	ΔG(CO)	ΔG(CHO)	ΔG(CHOH)	ΔG(CH)	ΔG(CH_2)	ΔG(CH_3)	ΔG(CH_4)	Overpot.
clean	-1.809	-2.139	-0.289	-2.612	-0.096	-1.987	-0.190	-0.416	-0.096
hrol	-1.219	0.330	-2.629	-1.533	1.485	-2.474	-2.428	-1.017	1.485
hdin	-1.771	-1.256	-1.227	-1.649	0.401	-2.105	-1.209	-0.614	0.401
hh	-0.611	0.273	-2.621	-0.170	-0.914	-1.402	-2.407	-1.374	0.273

Table S12. Gibbs energy for the CH_4 electrochemical path at 298.15K, V_{ext}=0.9 V, pH=5. All numbers are in eV. Column 4 produce in reality *CHO + *H_{rol} for the clean surface.

	1	2	3	4	5	6	7	8	
*Surface	ΔG(HCOO)	ΔG(CO)	ΔG(CHO)	ΔG(CHOH)	ΔG(CH)	ΔG(CH_2)	ΔG(CH_3)	ΔG(CH_4)	Overpot.
clean	-0.909	-1.239	0.611	-1.712	0.804	-1.087	0.710	0.484	0.804
hrol	-0.319	1.230	-1.729	-0.633	2.385	-1.574	-1.528	-0.117	2.385
hdin	-0.871	-0.356	-0.327	-0.749	1.301	-1.205	-0.309	0.286	1.301
hh	0.289	1.173	-1.721	0.730	-0.014	-0.502	-1.507	-0.474	1.173

Table S13. Gibbs energy for the CH_4 electrochemical path at 298.15K, V_{ext}=0 V, pH=5. All numbers are in eV. Column 4 produce in reality *CHO + *H_{rol} for the clean surface.

Thermal path was also investigated.

1. * + CO_2(g) + ½ H_2(g) → *HCOO
2. *HCOO + ½ H_2(g) → *CO + H_2O (g)
3. *CO + ½ H_2(g) → *CHO
4. *CHO + ½ H_2(g) → *CHOH
5. *CHOH + ½ H_2(g) → *CH + H_2O(g)
6. *CH + ½ H_2(g) → *CH_2
7. *CH_2 + ½ H_2(g) → *CH_3
8. *CH_3 + ½ H_2(g) → CH_4(g) + *

	1	2	3	4	5	6	7	8	
*Surface	ΔG(HCOO)	ΔG(CO)	ΔG(CHO)	ΔG(CHOH)	ΔG(CH)	ΔG(CH_2)	ΔG(CH_3)	ΔG(CH_4)	Overpot.
clean	-0.962	-0.761	0.559	-1.765	1.283	-1.140	0.657	0.431	1.283
hrol	-0.372	1.709	-1.782	-0.686	2.863	-1.626	-1.581	-0.170	2.863
hdin	-0.923	0.122	-0.380	-0.802	1.779	-1.258	-0.361	0.233	1.779
hh	0.236	1.651	-1.774	0.677	0.464	-0.554	-1.560	-0.527	1.651

Table S14. Gibbs energy for the CH_4 thermal path at 298.15K, Q=1/8, all energies in eV.

	1	2	3	4	5	6	7	8	
*Surface	ΔG(HCOO)	ΔG(CO)	ΔG(CHO)	ΔG(CHOH)	ΔG(CH)	ΔG(CH_2)	ΔG(CH_3)	ΔG(CH_4)	Overpot.
clean	-1.812	-0.616	0.488	-1.808	1.402	-1.152	0.525	0.245	1.402
hrol	-1.242	1.777	-1.790	-0.725	2.913	-1.720	-1.568	-0.187	2.913
hdin	-1.769	0.243	-0.431	-0.877	3.479	-1.248	-0.347	-0.216	3.479
hh	-0.657	1.716	-1.782	0.629	0.646	-0.777	-1.544	-0.513	1.716

Table S15. Gibbs energy for the CH_4 thermal path at 900K, Q=1/8 , all energies in eV.

	1	2	3	4	5	6	7	8	
*Surface	ΔG(HCOO)	ΔG(CO)	ΔG(CHO)	ΔG(CHOH)	ΔG(CH)	ΔG(CH_2)	ΔG(CH_3)	ΔG(CH_4)	Overpot.
clean	-2.001	-0.583	0.470	-1.822	1.431	-1.161	0.497	0.213	1.431
hrol	-1.434	1.799	-1.798	-0.739	2.931	-1.742	-1.573	-0.186	2.931
hdin	-1.957	0.273	-0.447	-0.896	3.880	-1.253	-0.346	-0.300	3.880
hh	-0.851	1.737	-1.790	0.614	0.686	-0.819	-1.548	-0.507	1.737

Table S16. Gibbs energy for the CH_4 thermal path at 1000K, Q=1/8, all energies in eV.

The optimal thermal path has been written in 8 steps in the manuscript to allow easy comparison with the electrochemical path. The adsorptions of H_2(g) can however be taken as separated steps.

1. *+ CO_2(g) + ½ H_2(g) → *COOH
2. *COOH + ½ H_2(g) → *CO + H_2O (g)
3. 2* + H_2(g) → + *H_{din} + *H_{rol}

4. $*CO + *H_{din/rol} \rightarrow *CHO$
5. $*CHO + *H_{rol/din} \rightarrow *CHOH$
6. $2* + H_2(g) \rightarrow + *H_{din} + *H_{rol}$
7. $*CHOH + ½ H_2(g) \rightarrow *CH_2OH$
8. $*CH_2OH+ ½ H_2(g) \rightarrow *CH_3OH$
9. $*CH_3OH + ½ H_2(g) \rightarrow *CH_3 + H_2O(g)$
10. $*CH_3 + ½ H_2(g) \rightarrow CH_4(g) + *$

	ΔG(1)	ΔG(2)	ΔG(3)	ΔG(4)	ΔG(5)	ΔG(6)	ΔG(7)	ΔG(8)	ΔG(9)	ΔG(10)
T=1000K	-2.001	-0.583	-0.254	-1.094	-0.057	-0.077	-0.921	-0.225	-0.536	-0.507
T=900K	-1.812	-0.616	-0.222	-1.094	-0.051	-0.055	-0.906	-0.262	-0.507	-0.513
T=298.15K	-0.962	-0.761	-0.106	-1.097	-0.019	0.004	-0.857	-0.442	-0.352	-0.527

Table S17. Gibbs energy for the CH_4 optimal thermal path with Q = 1/8, all energies in eV.

We see that the step 6 is blocking the reaction at 298.15 K.

**no H**		**E0 (eV)**	**TS (298.15K) eV**	**ZPE (eV)**	**TS(900K) eV**	**TS(1000K) eV**
**clean**	ncoo	-646.0407	0.2343	0.9566	2.1326	2.5553
**clean**	ncooh	-650.501	0.2392	1.3359	2.313	2.7818
**clean**	nco	-640.4451	0.1747	0.9072	1.8088	2.1801
**clean**	ncoh	-644.7097	0.1756	1.2265	1.904	2.3027
**clean**	nch1o	-643.4008	0.1954	1.2069	1.9691	2.3748
**clean**	nch1oh	-648.726	0.2038	1.5406	2.0902	2.5257
**clean**	nch1	-636.6128	0.1385	1.0969	1.6105	1.9528
**clean**	nch2oh	-650.9816	0.2091	1.8468	2.2014	2.6666
**clean**	nch2	-641.3224	0.1443	1.4372	1.6973	2.0646
**clean**	nch3oh	-653.6392	0.2602	2.0187	2.439	2.9408
**clean**	nch3	-644.14	0.1827	1.7152	1.9369	2.3481
**clean**	nch4	-647.0059	0.2526	1.8466	2.2619	2.7208
**H pyrrolic**						
**hrol**	ncoo	-652.0118	0.2333	1.2871	2.2128	2.6605
**hrol**	ncooh	-655.9607	0.2423	1.6735	2.4144	2.9106
**hrol**	nco	-643.3739	0.2034	1.2085	2.0114	2.4221
**hrol**	ncoh	-648.5443	0.1954	1.4153	1.9385	2.3408
**hrol**	nch1o	-648.7224	0.2036	1.54	2.0885	2.5238
**hrol**	nch1oh	-652.9436	0.2107	1.8474	2.2044	2.6695
**hrol**	nch1	-639.2102	0.1665	1.3852	1.8152	2.1977
**hrol**	nch2oh	-656.2373	0.2167	2.1766	2.3248	2.8201
**hrol**	nch2	-644.3555	0.1832	1.6856	1.9947	2.4147
**hrol**	nch3oh	-659.4222	0.2306	2.3574	2.3485	2.8472
**hrol**	nch3	-649.5255	0.1906	2.047	2.0581	2.4991

**hrol**	nch4	-653.0229	0.2242	2.1725	2.1774	2.6339
**H pyridinic**						
**hdin**	ncoo	-649.1347	0.2396	1.2642	2.2359	2.6867
**hdin**	ncooh	-655.6619	0.2353	1.6627	2.3816	2.8743
**hdin**	nco	-644.7092	0.1765	1.2258	1.9071	2.306
**hdin**	ncoh	-646.36	0.2059	1.4819	2.1263	2.5685
**hdin**	nch1o	-648.6171	0.1933	1.5353	2.0442	2.4746
**hdin**	nch1oh	-652.9454	0.2109	1.8438	2.2057	2.671
**hdin**	nch1	-640.4902	0.1478	1.4091	1.7403	2.1138
**hdin**	nch2oh	-655.9542	0.2138	2.1524	2.2879	2.7791
**hdin**	nch2	-645.324	0.146	1.748	1.7971	2.1924
**hdin**	nch3oh	-658.842	0.2303	2.3439	2.3437	2.842
**hdin**	nch3	-649.1791	0.1575	2.018	1.8629	2.2735
**hdin**	nch4	-652.2192	0.2777	2.176	2.5007	3.0115
**H both**						
**hh**	ncoo	-654.6493	0.2073	1.5929	2.1359	2.583
**hh**	ncooh	-660.1386	0.2484	1.9792	2.5277	3.0535
**hh**	nco	-647.6086	0.2111	1.5151	2.1301	2.5708
**hh**	ncoh	-650.8293	0.2185	1.7973	2.2635	2.7374
**hh**	nch1o	-652.944	0.2113	1.8416	2.2072	2.6726
**hh**	nch1oh	-655.827	0.2236	2.1789	2.3372	2.833
**hh**	nch1	-644.5931	0.1457	1.7831	1.7813	2.1737
**hh**	nch2oh	-660.2079	0.23	2.4742	2.4617	2.9891
**hh**	nch2	-648.5473	0.193	1.9949	2.12	2.5706
**hh**	nch3oh	-664.0614	0.222	2.6422	2.3423	2.8486
**hh**	nch3	-653.6875	0.199	2.3457	2.1786	2.6501
**hh**	nch4	-657.5628	0.224	2.4836	2.2579	2.7402

Table S18. Raw data for ground state energy (E0), zero point energy (ZPE) and entropic contribution (TS) at different temperature. The first column specifies the surface, clean is the basic 4N and a O cavity, hrol is the cavity with a pyrrolic H, hdin is the cavity with a H pyridinic and hh with both pyrrolic and pyridinc. The second column specify which molecule was attached.

Geometrical structures

All geometrical structures are available on the ioChem-BD database through the following link:

https://iochem-bd.iciq.es/browse/review-collection//fcf24395bc7810b48bf95bea
